# Palliative Care for SARS-CoV-2 Patients in the Intensive Care Unit: A Comprehensive Study

**DOI:** 10.1590/0034-7167-2023-0218

**Published:** 2024-06-28

**Authors:** Marilia Alves Furtado, Vitória Pessoa Nogueira, Maria Clara Passos Araújo, Virna Ribeiro Feitosa Cestari, Vera Lúcia Mendes de Paula Pessoa

**Affiliations:** I Universidade Estadual do Cearpa. Fortaleza, Ceará, Brazil

**Keywords:** Covid-19, Intensive Care Unit, Palliative Care, Sars-Cov-2, Terminal Care, Covid-19, Cuidado Terminal, Cuidados Paliativos, Sars-Cov-2, Unidades de Cuidados Intensivos

## Abstract

**Objective:**

To comprehend the multiprofessional actions regarding palliative care for patients in the Intensive Care Unit affected by SARS-CoV-2.

**Methods:**

A comprehensive qualitative study conducted with 31 professionals from the Intensive Care Units of a university hospital, based on the Theory of Peaceful End of Life.

**Results:**

The analysis of the discourse led to the identification of two categories: “Multidisciplinary actions to promote comfort at the end of life” and “Palliative care during the pandemic period and the promotion of emotional and spiritual comfort.”

**Final Considerations:**

It became evident that local administration needs to invest in measures that reduce barriers to the implementation of palliative care during times of crisis. Understanding the discourse highlighted that non-specialized professionals can provide basic palliative care appropriately, without diminishing the importance and necessity of the presence of palliative care specialists in various hospital areas.

## RESUMEN

**Objetivo:**

Comprender las acciones multiprofesionales en la atención paliativa de pacientes en una Unidad de Cuidados Intensivos afectados por el SARS-CoV-2.

**Métodos:**

Estudio integral y cualitativo realizado con 31 profesionales de Unidades de Cuidados Intensivos de un hospital universitario, basado en la Teoría del Final de Vida Pacífico.

**Resultados:**

El análisis de los discursos llevó a la identificación de dos categorías: “Acciones multidisciplinarias para promover el confort al final de la vida” y “Cuidados paliativos durante el período de la pandemia y el fomento del confort emocional y espiritual”.

**Conclusiones:**

Se evidenció la necesidad de que la administración local invierta en medidas que minimicen las barreras para la implementación de la atención paliativa durante los momentos de crisis. La comprensión de los discursos resaltó que los profesionales no especializados pueden proporcionar cuidados paliativos básicos de manera adecuada, sin desmerecer la importancia y la necesidad de contar con especialistas en Cuidados Paliativos en diferentes áreas hospitalarias.

## INTRODUCTION

Palliative Care (PC) is an approach aimed at enhancing the quality of life for patients and their families facing life-threatening illnesses or intense suffering related to health issues, with a focus on providing physical, psychosocial, and spiritual support^(1)^. Due to the nature of this care, the World Health Organization (WHO) considers its provision essential in situations of disasters and humanitarian crises, such as the SARS-CoV-2 pandemic. Despite the majority of efforts in such situations being focused on saving lives, measures to alleviate suffering should be considered, providing healthcare based on ethical principles^(2)^.

In the context of the recent SARS-CoV-2 pandemic, the limitation of the number of professionals in closed units and the need to conserve the use of personal protective equipment (PPE) were limiting factors for the effective presence of PC specialists in closed units, such as the Intensive Care Unit (ICU), leading to the necessity for this care to be primarily provided by healthcare professionals in those units^(3)^.

The organization of healthcare services to incorporate PC as a cross-cutting axis, especially in pandemic and disaster scenarios, is essential to ensure care based on ethical principles, comfort, and quality. The principles of PC are considered to contribute to the provision of comprehensive healthcare; however, their implementation in crisis situations and disruptions of the healthcare system becomes challenging^(4)^.

A scoping review study aimed at analyzing scientific evidence on the integration of PC during the SARS-CoV-2 pandemic did not find research conducted in the South American context^(4).^ A study conducted in the United Kingdom, on the other hand, showed an exponential increase in the number of severe SARS-CoV-2 patients. The number of patients referred to the PC team significantly increased, from two cases per week to 51 cases per week^(5)^.

In this way, enhancing healthcare choices is essential. Despite the losses during pandemic times being immeasurable, it becomes urgent to rethink how to face such challenges in future moments^(6)^. It is, therefore, essential to reconsider the practice of palliation provided by intensive care professionals during the pandemic period in favor of reflecting on the provision of such care in a scenario with limited specialist team involvement, providing a foundation for rethinking future coping strategies.

### Theoretical-Methodological Framework

In order to enable this reflection, it is considered essential to use a theoretical framework that supports palliative practice. The Theory of Peaceful End of Life (TFPE) is a valuable instrument due to its clear alignment with palliative principles and the practical nature of its assumptions, capable of guiding professionals’ attention toward effective palliative care^(7)^.

The theory outlines five assumptions for achieving a peaceful death, including: 1) absence of pain; 2) experiencing comfort; 3) dignity and respect; 4) closeness to significant people; and 5) being at peace. The TFPE supports the provision of healthcare that promotes the well-being of patients and their families in a multidimensional way, considering the complexity of the individual in the final phase of the life cycle^(8)^.

## OBJECTIVE

To comprehend the multiprofessional actions regarding palliative care for patients in the Intensive Care Unit affected by SARS-CoV-2.

## METHODS

### Ethical Considerations

The ethical aspects of research involving human subjects were upheld in accordance with Resolution 466/2012 of the National Health Council^(9)^. The study adhered to both national and international ethical guidelines and received approval from the Research Ethics Committee of the State University of Ceará, with the approval document attached to this submission. Informed consent was obtained in writing from all individuals involved in the study.

### Study Type

This is a comprehensive qualitative study guided by the COREQ tool^(10)^. Comprehensive studies focus on the subjectivity of social life, with the aim of understanding and interpreting the reality of human phenomena generated in society^(11)^.

### Methodological Procedures

####  Hypotheses 

It was hypothesized that non-specialist professionals were required to provide palliative care to patients in the units.

####  Study Setting 

The study was conducted in a large university hospital located in the city of Fortaleza, Ceará, during the months of June and July 2022. The setting included two Intensive Care Units (ICUs) designated for the care of critically ill patients affected by SARS-CoV-2. Each unit had eight inpatient beds, totaling 16 beds for the care of critical patients with SARS-CoV-2.

### Data Source

The research participants were members of the multiprofessional team working in the aforementioned hospital units during the SARS-CoV-2 pandemic. Professionals who were absent from their duties for more than 30 days at the time of data collection were excluded, as were those who were hired for a specific period to respond to the pandemic situation and whose contracts had expired. The subjects were identified by searching for the list of professionals working in the units during the pandemic period, obtaining this information from the team coordinators in their respective units.

Participants were invited via email, which contained information about the research, and they were required to sign the Informed Consent Form (ICF) in person upon acceptance. The initial search for subjects resulted in a population of 80 professionals. Of these, three were on vacation or on leave, and one reported not having worked in the units during the pandemic, resulting in the exclusion of four subjects.

In addition, 28 professionals declined to participate, and three agreed to participate but did not show up for their scheduled appointments and did not respond to further contact attempts. Of the remaining 43 professionals, subjects were successively included until data saturation was reached, and interviews were concluded after data collection with 31 professionals. Regarding participant characterization, nine were nurses, four were physiotherapists, four were physicians, one was a psychologist, and 13 were nursing technicians.

### Data Collection and Organization

The collection of discourses took place individually in a virtual format, using the Google Meet virtual meeting application, at a date and time agreed upon with the participant in advance. Semi-structured interviews were conducted by the principal researcher of the project, who holds a specialization degree in Intensive Care Nursing and has experience in qualitative research. Data collection was concluded when discursive recurrence was observed^(12)^. Initially, a questionnaire was administered, including questions about age, gender, profession within the unit, educational level, postgraduate education in the field of ICU, postgraduate education in the field of PC, courses or training related to PC during academic and/or professional education, prior work experience in the ICU, years of professional experience, years of experience in the ICU, and years of experience in PC. The interviews were recorded using the recording features of Google Meet.

### Data Analysis

The discursive content was transcribed in full, separately by the principal researcher of the project and a second researcher to enhance the validity of the process and for the subsequent organization of the discourses. The discursive corpus was prepared for submission and processing using the IRaMuTeQ program (R Interface for Multidimensional Text and Questionnaire Analyses). This software offers various methods for the treatment and analysis of textual data, including basic lexicographical analysis for word frequency and multivariate analyses, such as Descending Hierarchical Classification (CHD) and similarity analyses^(13)^.

For the statistical analysis of the discourses, the CHD method was chosen. This method classifies textual segments based on their vocabularies, aiming to obtain classes of Elementary Context Units (ECU) that share similar vocabulary among themselves and differ from other classes^(13)^. It should be noted that only words with statistically significant results, with a p-value of <0.001, were considered for the analysis. Based on the assumptions of the TFPE, the analytical categories identified through the CHD method allowed for the recognition of care actions and experiences of the interviewed professionals.

The comprehensive and interpretive process focused on the categories: “Multidisciplinary Actions for End-of-Life Comfort” and “Palliative Care During the Pandemic Period and the Promotion of Emotional and Spiritual Comfort,” as shown in Chart 1. Participants were identified by acronyms according to their professional category and the order of participation, to preserve the confidentiality and ethics of the research. The acronym “N” corresponds to nurses, “PH” to physiotherapists, “T” to nursing technicians, “PS” to psychologists, and “PHY” to physicians, with the numbering corresponding to the order of the interviews within each category.

**Chart 1:** Process of reducing the classes generated by the Descending Hierarchical Classification and creating analytical categories. Fortaleza, Ceará, Brazil, 2022

Lexographic Analysis			Classes (% of total)	Categories according to TPFE (Theory of Peaceful End of Life)
Word	X^2^	%		
To obtain To talk Doctor Time ICU To work To leave	32.98 29.95 28.52 25.67 25.11 24.75 15.21	61.90 100.00 46.15 47.06 62.5 100.00 58.33	1 - Challenges of palliation in the pandemic scenario (17.16%)	Palliative care in the pandemic period and the promotion of emotional and spiritual comfort (42.2%)
To play Music Catholic Family Day To ask To like Anointing oil Conscious To stay	62.33 57.04 40.34 34.23 32.09 26.22 24.20 23.96 21.84 16.23	73.33 100.00 100.00 44.44 83.33 71.43 80.00 100.00 62.50 34.62	4 - Emotional and spiritual comfort in the face of pandemic difficulties (11.27%)	
Visit Infirmary Family Death Cry Person Contact Moment Release Cell phone Exist	45.56 38.86 36.12 25.65 25.65 20.38 20.38 19.29 19.14 19.14 16.73	100.00 69.23 69.23 100.00 100.00 71.43 71.43 50.00 100.00 100.00 62.50	6 - Approach between individuals in illness and dying during the pandemic period: the reintegration of spirituality and individuality of individuals (13.73%)	
Palliative Care Action Reiki Question Massage	83.29 81.32 20.40 16.66 15.22	71.88 73.33 100.00 45.83 100.00	2 - Attention to spiritual comfort (16.67%)	Multidisciplinary actions for end-of-life comfort (57.8%)
Maximum Try Palliative Nursing Maintain People Measure	39.00 30.18 29.31 28.89 24.46 17.40 16.73	80.00 66.67 46.43 43.75 63.64 47.06 62.50	3 - The multiprofessional effort to relieve physical suffering (13.73%)	
Continue Change of position Mute Care Hygiene Change Alone Nursing	98.98 47.45 44.26 38.70 36.70 21.88 17.83 17.27	93.33 64.71 100.00 42.11 100.00 80.00 46.67 34.38	5 - No change in the nursing care routine and attention to physical aspects of care (12.25%)	
Physiotherapy Mode Aspiration Secretion Ventilator Uncomfortable Remove Condition	96.21 22.77 22.77 22.77 16.99 16.99 16.70 16.70	94.44 100.00 100.00 100.00 100.00 100.00 80.00 80.00	7 - Attention to physical aspects of care and limitation in the use of invasive painful measures (15.2%)	

*X^2^: chi-squared.*

## RESULTS

As for the social and professional aspects of the participants, 24 (77.4%) were female, with an average age of 39.4 years. Regarding their educational background, the majority had completed their undergraduate degree (90.3%). Only two (6.4%) participants had specialized training in PC, and 20 professionals (64.5%) reported the absence of PC-related courses during their undergraduate or postgraduate studies, as illustrated in Table 1. It’s worth noting that, even though most participants didn’t have specialized training or specific education in PC during their academic programs, they mentioned a connection with the PC specialist team due to the team’s extensive involvement in the hospital before the pandemic.

**Table 1:** Sociodemographic characterization of the subjects included in the research. Fortaleza, Ceará, Brazil, 2022

Participant Characteristics	n (%)
Gender	
Female	24 (77.4)
Male	7 (22.5)
Age (years)	
Mean±SD (Standard Deviation)	39.38±6.1
Minimum	29
Maximum	54
Highest Education	
Technical level	3 (9.6)
Undergraduate	10 (32.2)
Postgraduate	8 (25.8)
Master's	8 (25.8)
Doctorate	5 (16.1)
Specialization in Palliative Care	
Yes	2 (6.4)
No	29 (93.5)
Palliative Care Discipline or Course in Professional Training	
Yes	11 (35.4)
No	20 (64.5)

**Figure 1:**
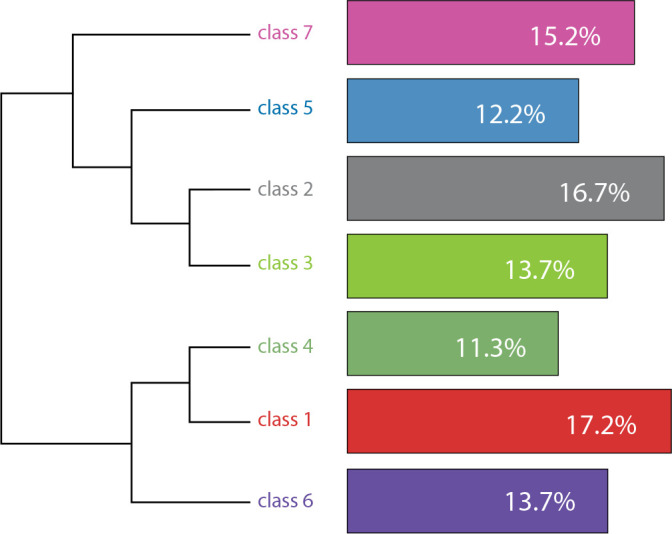
Dendrogram of the classes provided by IRaMuTeQ. Fortaleza, Ceará, Brazil, 2022

After processing the corpus in the text analysis software, an efficiency of 91.5% was achieved with the submitted material. The LDA made it possible to identify seven classes generated by the software (Figure 1).

Classes two, three, five, and seven were merged as they contained statements primarily related to professionals’ emphasis on the “absence of pain” and, to a lesser extent, the “being at peace” assumption, constituting 57.8% of the content. This category was named “Multidisciplinary Actions for End-of-Life Comfort.”

Subsequently, classes one, four, and six were consolidated due to their similarities, encompassing statements that represented professionals’ strategies for overcoming the challenges of implementing palliative care during the pandemic. These statements also embraced the assumptions of “experiencing comfort,” “dignity and respect,” “closeness to significant people,” and “being at peace.” This second analytical category comprised 42.2% of the discourses and was named “Palliative Care During the Pandemic Period and the Promotion of Emotional and Spiritual Comfort.”

The following results will be presented based on the emerging categories following the comprehensive movement.

### Multidisciplinary Actions for End-of-Life Comfort

Understanding this analytical category revealed the predominance of care actions that prioritized the “absence of pain” assumption, as demonstrated by the professionals’ concern in optimizing the use of pharmacological measures for pain relief, with an emphasis on participants from the nursing and physiotherapy categories.

*You know, it was about comfort, analgesia, you know?* (N08)

*The main things we used and were using at the time were about pain control, right? A lot of pain control.* (PH03)

Some professionals from the technical nursing team reported the use of non-pharmacological strategies and alternative therapies for anxiety relief and the promotion of patients’ spirituality. This revealed the utilization of the “being at peace” assumption of the TFVP, as shown in the following statements.

*I used to do massages. I used Reiki, said prayers... I did a lot of hands-on, and for the patients who were more emotionally debilitated, depressed, or even anxious, I used Reiki a lot.* (T10)

### Palliative Care During the Pandemic Period and the Promotion of Emotional and Spiritual Comfort

This category facilitated the understanding of the challenges in integrating the PC team into the critical care of SARS-COV-2 patients beyond the actions mentioned.

*There was no palliative care. It was more or less closed off by the doctor, the head of the ICU, and the ICU medical team. There was no participation from the palliative care team in the Covid ICU.* (N09)

One of the reasons cited for this was the increased workload of the PC specialist team.

*...we didn’t manage to have the participation of the palliative care team as frequently as we would have liked... because the demand was very high in other areas of the hospital too.* (PHY04)

Another reason reported by professionals for the reduced involvement of the PC team in the sector was the issue of limited PPE access, as well as restrictions due to infection risk.

*I think that was because of the initial access difficulty to patients. It was complex due to the material...* (N02)

Moreover, the fear of the unknown, due to the new clinical condition, created more resistance among professionals in engaging the palliative care team.

*The fact that the disease was very new, palliative care was only for Covid, and there was nothing else, so when we called, it was already very late. And often, it was sort of reluctantly. There wasn’t unanimous agreement from everyone.* (PHY02)

However, despite these challenges, the discourse content indicates that the absence of the specialist team motivated the healthcare professionals in the units to take full responsibility for providing PC.

*...in many cases, we, as an ICU team, had to take full responsibility for this issue. There was more autonomy in this regard. We could intervene more early with the families since we could perceive the clinical condition’s irreversibility and severity.* (PHY04)

The understanding of the participants’ discursive content reveals that despite the difficulties in integrating PC during the pandemic, the multiprofessional team made efforts to provide such care. They used strategies based on palliative principles.

The mentioned PC actions show a patient-centric approach aimed at relieving suffering caused by the pandemic. They provide actions based on the assumption of “experiencing comfort” by promoting emotional and spiritual comfort, as well as actions based on the “closeness to significant people” assumption by fostering contact between the patient and their family to overcome the social isolation during this period.

*We would attend to the patient and make significant efforts to establish an emotional connection between the patient and their family through video calls.* (PS01)

*We realized that it brought comfort and relief. It allowed the family to get closer. It even allowed the family to visit, even with Covid. It provided that possibility because when palliative care arrived, we already thought about letting the family in.* (PHY02)

Respect for personal beliefs was mentioned, including the inclusion of religious rituals as requested by families and patients, demonstrating the application of the “dignity and respect” and “being at peace” assumptions outlined in the TFVP.

*We respected the patients’ religious beliefs... There was a doctor who included in the prescription: holy water, three times a day. Anointing oil, two times a day. He wrote it as the family requested, and put it in the patient’s care... I’m a Catholic, but I have to respect other people’s religion.* (N09)

The above discourse demonstrates the use of strategies to strengthen weakened bonds and provide actions for the restructuring of patients’ mental and spiritual health in intense suffering. These actions maintain the dignity of the individuals and respect their beliefs, practically applying the assumptions outlined in the TFVP.

## DISCUSSION

The results of this study reflect the complexity of palliative care in the context of the SARS-CoV-2 pandemic and the actions taken by healthcare professionals. First and foremost, the study demonstrates the presence of various barriers to the implementation of palliative care during the pandemic. Similar difficulties were reported in a study conducted in Canada, which showed that infection control measures and restrictions on the use of PPE and the number of professionals in ICUs were barriers to the effective presence of palliative care professionals in units^(3)^, corroborating the findings of this study.

A study conducted in Denmark, comparing the referral rate of patients to the PC service before and after the SARS-CoV-2 pandemic, found a decrease in the activation of the palliative care team. These findings, along with those of the current study, highlight the need for local health service management to act in a way that minimizes these barriers. This can be achieved by allocating resources to enable palliative care, such as hiring specialized professionals to meet the high demand of patients with palliative needs. Investment in continuing education for healthcare professionals on palliative care is also necessary to maintain the quality of care for patients facing life-threatening situations^(14)^.

Despite the barriers posed by the pandemic, the comprehensive understanding of the discourses revealed that the multiprofessional ICU team made efforts to offer care focused on relieving the suffering of patients with palliative needs. The study demonstrated a connection with the assumptions of the TFPE in the care provided by non-specialists in palliative care. This provision of palliative care by healthcare teams is already recommended by the National Academy of Palliative Care (ANCP), recognizing especially ICU professionals as capable of meeting most of their clients’ palliative care demands^(15)^. However, this does not eliminate the need for local management to ensure the presence of palliative care specialists in hospital units to provide assistance in addressing the special and complex needs of patients.

In a study conducted in an ICU in the state of Bahia, which was based on the TFPE as a theoretical framework, the authors found that all members of the multiprofessional team considered pain relief as a fundamental aspect of patient palliative care. Additionally, the participants revealed the importance of involving the family in the care of patients in the terminal phase of illness^(16)^, which aligns with the results found in the present study.

When considering patient care for pain, however, the current concept of pain defined by the International Association for the Study of Pain (IASP) should be taken into account. Pain is defined as an unpleasant sensory and emotional experience typically caused or resembling actual or potential tissue damage. The association acknowledges the subjective nature of pain, which can be influenced by physical, psychological, and social factors^(17)^. Thus, professionals should pay attention to aspects beyond the physical manifestation of pain, considering its complexity and multifactorial nature.

In addition to pain management, the multidimensional experience of comfort prominently appeared in the participants’ discourses. According to the TFPE, the experience of comfort involves relieving any discomfort, a state of tranquility and contentment, or anything that makes life pleasant^(8)^. The participants’ discourses demonstrate attention to comfort through concerns about analgesia, positional therapy, avoiding frequent use of painful methods, as well as concerns about oxygen therapy and bodily aesthetics. Moreover, the use of alternative therapies such as Reiki demonstrates a focus on multidimensional comfort, as advocated by the TFPE. Furthermore, the use of alternative and complementary therapies is a recommended practice by the Ministry of Health, which recognizes that these approaches seek to stimulate natural mechanisms for health restoration through an expanded view of the health-disease process and the promotion of humanized care and should be encouraged by healthcare managers^(18)^.

On the other hand, the use of religious practices, as well as the provision of actions for promoting spirituality, as demonstrated in the participants’ discourses, shows an alignment with the assumptions of “dignity and respect” as well as “being at peace.” A qualitative study based on the assumptions of the TFVP demonstrated that nurses value spirituality as a resource for promoting peace through the inclusion of religious practices^(7)^.

Furthermore, the assumption of “closeness to significant people” stood out significantly when understanding the professionals’ concern in facilitating the connection between loved ones and overcoming the social distancing imposed by the pandemic. According to the TFVP, this assumption involves the feeling of closeness and connectivity with other individuals considered important by the patient. In a broader perspective, it means enabling family involvement in care^(8)^. A review study on providing palliative care to seriously ill patients during the pandemic period demonstrated that the majority of studies point to the use of strategies for connecting patients with their families, primarily through virtual visits, which can help alleviate the impact of the social distancing measures during hospitalization^(19)^.

### Study Limitations

This study has some limitations that should be discussed. First, it is important to mention the high number of refusals by subjects invited to participate in the research. This highlights the persistent complexity involved in conducting qualitative research, as it necessitates ensuring the active and voluntary participation of the interviewees.

Additionally, the research was conducted in a tertiary hospital that has specialized teams for providing palliative care. Consequently, the interviewed subjects have considerable experience in the field of palliative care due to the nature of their daily activities. This expertise may have influenced the approach and actions related to palliative care in the context of the pandemic, potentially making these results not representative of the reality found in other healthcare institutions in the national context. Therefore, it is essential to consider the possible variability among healthcare institutions that may not have the same degree of specialization in palliative care, which can impact the availability and quality of palliative care provided to Covid-19 patients.

### Contributions to the Nursing, Health, or Public Policy Fields

It is believed that knowledge about the provision of palliative care by non-specialist healthcare professionals during the SARS-CoV-2 pandemic can facilitate the development of strategies to address future catastrophic situations and disruptions in the healthcare system while maintaining palliative care for patients enduring intense human suffering in such scenarios. As the results of this study indicate that professionals from different areas had to be directly involved in providing palliative care, they also underscore the need to integrate palliative care into health policies at all levels. This may involve establishing interdisciplinary teams or revising policies to ensure that patients with palliative needs receive appropriate care, regardless of the emergency situation.

## FINAL CONSIDERATIONS

Our data highlight the necessity for local healthcare service management to invest in actions aimed at mitigating barriers to the provision of palliative care during healthcare system disruptions. This can be achieved by ensuring the presence of specialist professionals in the units and through continuous investment in Continuing Education on palliative care. However, despite the barriers imposed by the pandemic, the healthcare teams in the ICUs demonstrated the use of multidimensional strategies for the effective delivery of relief actions for patients with palliative needs, aligning with the Theory of Peaceful End of Life.

In this context, it is understood that non-specialist teams are capable of providing basic palliative care adequately, but the presence of palliative care specialists is essential to address the special and complex needs of patients. Furthermore, the value of palliative care actions for quality and patient-centered healthcare is demonstrated.

## Supplementary Material







## Data Availability

https://doi.org/10.48331/scielodata.NIZRCM
