# Supplementary material for: Palliative Care for SARS-CoV-2 Patients in the Intensive Care Unit: A Comprehensive Study
Source: Rev Bras Enferm. 2024 Jun 28;77(Suppl 1):e20230218. doi: 10.1590/0034-7167-2023-0218 (PMC11213540; doi:10.1590/0034-7167-2023-0218)
Supplement: Supplementary file 1 [file 0034-7167-reben-77-s1-e20230218-suppl1.pdf]

DADOS QUALITATIVOS

TRANSCRIÇÃO DAS ENTREVISTAS

PARTICIPANTE ENF 01

As ações de cuidado paliativo eram mais voltadas para não fazer nenhum tipo de procedimento muito invasivo. Suspendia os exames laboratoriais. A prescrição era reduzida a somente o estritamente necessário. Hidratação e conforto. Morfina. Da equipe de enfermagem os cuidados não mudavam, eles continuavam. Para a equipe de enfermagem não muda muita coisa, porque o paliativo é um cuidado de alta dependência. A equipe de enfermagem vai prestar o cuidado igual a de um paciente com bom prognóstico de alta dependência. Não muda o fato de ser paliativo, o cuidado continua. A enfermagem precisa até ter mais cuidado. Com a mudança de decúbito, colchão pneumático, cuidado com a pele, as punções. Os técnicos de enfermagem também têm um olhar sobre a necessidade do paciente paliativo fazer glicemia. Também questionam se não vai fazer mais nada. Uma dúvida que sempre fica na equipe de enfermagem é sobre o paliativo proporcional. Esse paciente é paliativo, mas é proporcional. O proporcional é o que faz tudo, todo exame laboratorial, todas as medicações, antibiótico, dialisa, mas não entuba e não reanima. Isso acontece muito. Porque se é paliativo, é paliativo. Se não é paliativo, não. Existe muito caso de paliativo que faz tudo, mas não reanima.

PARTICIPANTE ENF 02

As ações de cuidado paliativo foi muito de acompanhamento da psicologia com os familiares, principalmente de pacientes jovens, sem comorbidade e muito grave. Foi muito importante quando eles começaram a ter mais acesso ao acompanhamento da psicologia, aos cuidados paliativos, à equipe de cuidado paliativo e do serviço social. Toda a equipe de cuidado paliativo, que fornece apoio psicológico e apoio estrutural para as famílias. Mais para a família do que para o paciente, para a família ter acesso àquele doente. Os filhos, as esposas, os maridos. Foi mais a questão de cuidado com a família. A questão da psicologia era bem ativa, mantendo o cuidado com a família e com o paciente, todo o tempo. A enfermagem agia fora da uti. A enfermagem agia com a equipe multiprofissional, mais nas questões das reuniões, da limitação ou até de entender os pacientes mesmo. A enfermagem eu não via mudança de cuidado, porque a enfermagem continuava administrando medicações, fazendo os cuidados de enfermagem diários. Na mudança de perfil o paciente não deixa de continuar necessitando dos cuidados. A única diferença é a questão da limitação terapêutica, quando ele progride a gravidade. Mas a questão do cuidado em si não muda.

PARTICIPANTE ENF 03

As ações de cuidado paliativo foram importantes primeiro para a equipe de enfermagem entender o que podia ser feito com o paciente de cuidados. A enfermagem continuou tendo todos os cuidados de assistência para a higiene. De medicação teve algumas privações. Medicações para dor a gente fez muita. A gente a priorizar mais essas medicações para dor nesse contexto. E também o mais importante foi a inserção da família. A partir dos cuidados paliativos, a família pôde viver o seu ente querido. O serviço, na verdade, quando tem a reunião de cuidados paliativos, a família precisa estar inserida nesse processo, para a definição do quadro, definição das limitações, do tipo de cuidado paliativo. A partir disso, era estabelecido com a família que eles poderiam visitar, ter um momento do dia com o seu ente querido. O serviço social também estava inserido nesse processo. O serviço social ligava para a equipe do plantão pra saber se o familiar poderia ter aquele momento de visita. Era liberado visita, no horário da tarde. Quando era mais de uma pessoa da família, a enfermagem dividia o horário. A equipe de enfermagem tentava manter o ambiente um pouco mais privativo. Na uti covid existiam umas cortinas que isolavam os leitos. A enfermagem deixava o paciente o melhor possível aparentemente. Ele já tinha sido higienizado, banhado de manhã, com todos os cuidados de hidratação. Antes da visita, a enfermagem deixava o paciente numa posição onde favorecesse mais o contato do familiar com o paciente, e como era na uti covid, o familiar entrava todo paramentado, com todos os equipamentos de proteção individual, e geralmente ia um membro da equipe de enfermagem. Geralmente técnico de enfermagem ou enfermeiro, e acompanhava até o leito. Eu muitas vezes acompanhei, e eu falava que poderia ter contato, que falasse perto do ouvido, que qualquer coisa chamasse a equipe. A enfermagem fechava a cortina, para tentar dar um pouco mais de privacidade, e a pessoa poder de fato se entregar naquele momento durante a visita.

PARTICIPANTE ENF 04

As ações de cuidado paliativo eram decididas a partir de um plano de cuidados para o paciente. A não reanimação seria um plano de cuidado. Medida de conforto estava escrito na prescrição. A equipe se comunicava entre si. Na prescrição de enfermagem eram colocados alguns cuidados, mais especificamente. A equipe ficava sensibilizada. Você via a equipe mais sensibilizada. Quando ia uma família, a equipe avisava que a família ligou, que queria saber notícia do paciente. A enfermagem avisava ao médico pra fazer a ligação, porque ele fazia uma vez por período, que era de tarde, para passar informação para família. A enfermagem ficava cobrando. A equipe percebia um engajamento e uma tentativa dar o máximo, de não deixar descuidado o paciente. E a enfermagem fica o tempo todo falando pro médico que o paciente está com dor, que ele está só com morfina de resgate, fixo, sugerindo botar uma bomba de morfina. A equipe ficou um pouco mais sensibilizada.

PARTICIPANTE ENF 05

A ações de cuidado paliativo eram de limitação terapêutica, limitação de medidas invasivas. A equipe só deixava os métodos não invasivos no paciente. Sinais vitais ficavam de 6 em 6 horas, não colocava de 2 em2 horas. Para demonstrar que o paciente de algum modo estava sendo

assistido. O que a gente observava era o pensamento de que se é paliativo, não vai fazer mais nada. Mas o cuidado tem que continuar. A questão da higiene corporal, as medicações necessárias para dor. Até hoje eu observo paciente consciente, nem ele é avisado de que não vai ser feito nada. Eu vejo um medo do profissional médico, no geral, de profissional da saúde, de chegar e dizer que não vai mais ter benefício, e passar para a família.

PARTICIPANTE ENF 06

As ações de cuidado paliativo eram praticamente medidas de conforto. Manter analgesia, sonda vesical de demora, não colher culturas, não transportar para exame, não iniciar esquema novo de antibiótico, não aumentar vazões de drogas. Praticamente o que é feito na UTI que também não é Uti COVID. Não teve diferença. Embora o paciente fosse diferente. Mas sem dúvida, a gente começou a pensar em acompanhamento pelo paliativo e em paliar de fato no COVID muito mais tardiamente do que no paciente não COVID. Embora as condutas fossem as mesmas.

PARTICIPANTE ENF 07

As ações de cuidado paliativo na época eram o conforto, conforto de analgesia. Evitar o avanço de procedimentos invasivos. Basicamente isso. Mais para os pacientes mais limitados terapeuticamente, para esses pacientes que eram COVID com mais limitação terapêutica. Em relação aos demais pacientes clínicos, não. A equipe da noite acaba recebendo uma descontinuidade de algumas informações. O máximo que a equipe da noite sabia era que o paciente era paliativo e limitação terapêutica. A equipe da noite sabia que aquele paciente era mais para conforto.

PARTICIPANTE ENF 08

As ações de cuidado paliativo era de manter o conforto do paciente, porém ainda fazendo as medidas. Tinha muito o foco respiratório, e os cuidados eram os mesmos, mas sempre se realmente tivesse um quadro de paliação exclusivo, a gente dava o conforto e fazia todas as medidas para o conforto do paciente, nunca tendo aquela visão de que se é paliativo, não faz nada. A enfermagem sempre ficava priorizando o conforto, o cuidado. Não tinha diferenciação porque era paliativo. A enfermagem dava o mesmo cuidado. O que seriam as medidas de conforto? Analgesia. A enfermagem observava muita fácies de dor. Se o paciente tinha muita fácies de dor a gente procurava alguma coisa prescrita pra dor. A enfermagem estava sempre atenta à mudança de decúbito. Os cuidados de enfermagem em geral, que pudesse evitar o máximo de sofrimento. Não visualizar o paciente e porque é paliativo pensar que é fim de vida. Não é diferente dos outros. Ter o cuidado, o conforto melhor para o paciente. Sempre tendo uma visão holística e não diferenciando por ser paliativo e fazer menos coisas pra ele. Têm as medidas restritivas para cada caso. Um paciente realmente terminal, avaliar se coloca uma bomba de morfina pra ele não sentir dor e realmente chegar ao fim de vida tranquilo.

Avaliando. Se tem uma úlcera extensa, vale a pena ele realmente em fim de vida aplicar um curativo que precise de vários dias para progredir? Esse tipo de avaliação que a gente faz.

PARTICIPANTE ENF 09

As ações de cuidado paliativo do lado da enfermagem, eu acho que foi a equipe mais a humanizada, apesar de os médicos também serem. A equipe de enfermagem respeitava muito a questão das crenças religiosas dos pacientes. O santo era liberado, o terço era liberado, o papel de reza era liberado. Uma vez entrou um padre e um pastor. Os dois foram liberados a visita, porque os pacientes não tinham mais prognóstico. Era o que a família pedia muito. E os dois chegaram na mesma hora. Então eu disse que ia entrar os dois, os dois respeitando a religião de cada um e todos os dois iam fazer um culto ecumênico, vestindo todas as roupas do protocolo. Eles se entenderam e entraram. Foi um momento muito bonito, onde a equipe de plantão chorou muito. A questão espiritual do paciente é muito importante. Outro caso a equipe de enfermagem aperreou muito para o médico liberar a visita, porque não tinha mais prognóstico, e tinha um filho dele que fazia muito tempo que ele não via, para liberar o filho. A pessoa se responsabiliza. Ele se responsabiliza, veste todos os equipamentos de proteção individual. Ele entrou, falou com o pai dele, chorou. Foi uma situação de 20 a 30 minutos, um momento muito emocionante da despedida. Tinha um médico também que colocava na prescrição a água benta, 3 vezes ao dia. Óleo da unção, 2 vezes ao dia. Do jeito que a família pedia, ele colocava lá para colocar no paciente esses cuidados. As famílias traziam o óleo da unção. O óleo da unção é dos evangélicos. Eles têm um óleo da unção. Os evangélicos têm. Os católicos é água benta. Eles traziam e pediam que a água benta fosse colocada de manhã, à tarde e à noite. O óleo da unção era pela manhã e pela noite, quando ele fosse dormir. Esse único médico tinha esse cuidado. Só no dia dele ele colocava, para os técnicos e os enfermeiros fazerem o aprazamento. Eu achava de uma humanidade incrível. Porque não é todo médico. Ele pedia para as famílias mandarem mensagem de voz e a equipe colocava no ouvido do paciente, para o paciente escutar, e os pacientes escutavam, porque é o último sentido que o paciente perde. E alguns choravam, outros ficavam mais agitados com a voz. A equipe de enfermagem tentava fazer essas coisas quando era possível e que o médico aceitava. A equipe liberava as visitas, na medida do possível. Porque a espiritualidade é uma coisa que ajuda muito o paciente e a família a aceitar, mais ou menos, essa passagem da vida para a morte. É a primeira coisa que eles pedem. Esse médico tinha essa delicadeza que eu achava incrível dele. E o enfermeiro aprazava. Era o médico que mais liberava visita. No dia dele a enfermagem se organizava que era o dia que ele mais liberava visita dos familiares, que os pacientes estavam ruins. A dignidade, quando já via que realmente não tinha, eles diminuíam a prescrição, tentavam fazer menos distanásia possível. Tentavam manter a dignidade. Mas foi muito doloroso. A equipe tentava manter essa dignidade ao máximo desse paciente. Fazer as vontades dele. Os que estavam conscientes. Era muito complicado. Os médicos, a equipe toda, a equipe do hospital é muito boa. Tentava manter essa dignidade do paciente. Algumas vontades tentava fazer. Alguns desejos deles. Mas era complicado. Era muito complicado.

PARTICIPANTE FISIO 10

As ações de cuidado paliativo eram de priorizar medidas de conforto. Medidas de conforto. Uma aspiração. De atendimento quando a fisioterapia via, não fazia muita coisa não. É porque geralmente quando o paciente está em paliativo é mais medidas de conforto, por toda a equipe de plantão. Como era uma demanda muito grande de pacientes, foi muito difícil, muito conturbado. O tempo era muito corrido, e os pacientes de cuidados paliativos, geralmente, eram medidas de conforto. O que a fisioterapia fazia mais era manter o paciente confortável na ventilação mecânica, sem secreção.

PARTICIPANTE FISIO 11

As ações de cuidado paliativo pela parte da fisioterapia, que vê muito essa parte da respiratória, é o conforto. Não precisa estar fazendo higiene brônquica de horário, como a fisioterapia costuma fazer. Não é necessário a fisioterapia fazer a higiene brônquica. Porque o método de aspiração é o procedimento mais doloroso, dentro de uma uti, é a aspiração. De imediato, é só aspirar sobre demanda. Se o paciente estiver realmente muito desconfortável por secreção, a fisioterapia aspira. Se não, se o paciente estiver bem acoplado na ventilação mecânica, a ausculta estiver limpa, a fisioterapia não vai ver se tem secreção. E outra coisa também é o modo ventilatório que, claro que um paciente pode muito bem ficar bem em pressão de suporte, como eu já vi pacientes aqui agora em paliação, fica muito confortável em pressão de suporte, mas eu acho bem confortável a fisioterapia dar essa ventilação para ele. Deixar em modo assistido controlado. Não deixar o paciente que está em paliação Exclusiva entrar na ventilação, mesmo que ele fique muito confortável em pressão de suporte. Eu acho mais prudente deixar ele em modo assistido controlado. E manter conforto em relação a medidas de motora também, a fisioterapia motora. Tem alguns profissionais que ainda gostam da fisioterapia motora. Eu não vejo tanto sentido. É olhar para aquele paciente não de uma forma geral, mas para aquele paciente e ver o que é que a fisioterapia pode fazer agora, no estado de paliação, para manter o conforto. Se for só pra deixar ele em assistência, monitorização respiratória, é isso o que a fisioterapia vai fazer. De modo individualizado.

PARTICIPANTE FISIO 12

As ações de cuidado paliativo que a fisioyterapia usa e usava na época era a questão de controle de dor, muito controle de dor. Os pacientes usavam muito medicamento analgésico e até bloqueador neuromuscular. Muito bloqueador neuromuscular. Controle de dor, desconforto respiratório. Bastante isso. E depois a questão do medicamento. Fazia o desmame de alguns medicamentos que não havia necessidade naquele paciente mais. Diminuía a prescrição. Visava mais o controle dos sintomas. Na fisioterapia, como os pacientes eram bastante difíceis de ventilar, tentava ao máximo retirar a assincronia do ventilador. O paciente quando ficava com assincronia, brigando com o ventilador, causava desconforto respiratório. Com esse desconforto respiratório tinha taquipnéia, taquicardia, hipertensão. A fisioterapia fazia o máximo para não ter essa briga com o ventilador. Diminuir, ou até tirar esse

desconforto respiratório. Era o básico no covid. Era fazer isso com o paciente. A fisioterapia não podia manipular muito, porque eram pacientes muito críticos. Principalmente no paliativo, que é o final da vida. Se não conseguisse no ventilador melhorar as curvas, aí chamava o médico para aumentar a sedação, analgesia. É mais basicamente no ventilador. Nos programas do ventilador que a fisioterapia atua bastante. E também secreção, se for paciente entubado. Se o paciente estiver desconfortável, faz uma aspiração, uma técnica de remoção brônquica, de alguma secreção que gera também desconforto. Através de técnicas manuais e também assincronia com o ventilador. Da fisioterapia são essas coisas. Ver a questão do desconforto respiratório do paciente. Ver se o paciente não estiver entubado, se precisa de uma ventilação não invasiva, para tirar o desconforto respiratório. Com oxigenioterapia. São essas medidas que a gente pode atuar, para melhorar essa condição clínica do paciente.

PARTICIPANTE FISIO 13

As ações de cuidado paliativo normalmente são os mesmos. A enfermagem sempre tem o cuidado de dar o banho no paciente, de ficar trocando os acessos, de mudar o decúbito, para evitar ter uma lesão por pressão. Na ventilação mecânica, a fisioterapia tem o cuidado de evitar também de estar aumentando a FiO_2. Porque às vezes eles pedem para deixar sem aumentar. Não que vá deixar o paciente desconfortável, que piore. Mas não vai fazer ajustes para aumentar o nível de IPAP e EPAP, porque não vai ajudar em nada. Procurar deixar os parâmetros naquele mínimo possível que o paciente conseguir ficar e sempre monitorando. A fisioterapia faz a parte dela de estar atendendo. Tudo o que a gente faz com os outros pacientes, a gente faz com o paliativo. A diferença é mais, no meu modo de pensar, em relação a essas coisas invasivas. De reanimação, de não reanimar. De não entubar. Alguns casos de não dialisar. Eu acho que é mais nessa parte. Continua normal. Só no limite do paciente. Se for um paciente que está com febre, uma febre persistente, eu não vou fazer fisioterapia motora no paciente. Se for um paciente que já está ali dessaturando, que está bradicárdico, está com a frequência cardíaca baixa. O máximo que a fisioterapia vai fazer é um posicionamento no leito, uma aspiração para deixar o paciente mais confortável. Não vai ficar fazendo fisioterapia motora, querendo sentar o paciente. A não ser que seja um paciente em cuidados paliativos que seja consciente. Tem pacientes que andam, que estão conscientes que não tem mais o que fazer, por conta de um câncer que está cheio de metástase. Esses pacientes a fisioterapia ainda senta, ainda faz a parte da fisioterapia motora dele. Mas pela fisioterapia, não há muita diferença não. A gente atende.

PARTICIPANTE MED 14

As ações de cuidado paliativo eram assim, em se definindo que o paciente teria indicação de limitação terapêutica, aí você teria que individualizar. Vamos dizer que houvesse um paciente com comprometimento pulmonar extenso, que não conseguia sair da ventilação mecânica, que você via que aquele pulmão hepatizava mesmo e você via que o paciente não ia conseguir ter uma qualidade de vida com a retirada de suporte. Às vezes nem ia conseguir sair do suporte. Nesses pacientes especificamente o médico começava a indicar limitação de cuidado.

Evitava aumentar FiO2, não iniciava droga vasoativa, não rodava antibiótico. Conversar com a família, para explicar a situação que, às vezes, era até paciente jovem que estava nesse contexto. A eleição do paciente, e em si elegendo, individualizar um plano de cuidado, porque eu não gosto muito dessa questão de paliativo exclusivo, paliativo predominante não. Mas individualizar um plano de cuidado. Paciente não tem mais indicação de terapêutica modificadora de doença, então a equipe vai iniciar aqui um cuidado priorizando conforto. Paciente vai evoluir nas próximas 48 horas, então realmente o médico vai tirar todo suporte que ta prolongando o morrer. O paciente está naquela condição intermediária, que não sei realmente se ele vai evoluir desfavorável, mas se ele permanecer vivo ele vai ter uma qualidade de vida muito ruim. Tem que contextualizar, conversar com a família, avaliar e, às vezes, depois até da primeira conversa que o médico vai conseguir em um segundo momento limitar terapêutica.

PARTICIPANTE MED 15

As ações de cuidado paliativo, no começo, ninguém teve muita coragem de limitar terapia nos pacientes, mesmo muito grave e com prognóstico muito reservado, porque você tinha sempre um paciente ou outro, que embora muito grave, conseguia melhorar, apesar do acometimento grave demais, muito importante. Quando houve limitação terapêutica, quando tinha indicação de limitação terapêutica, o médico chamou o paliativo pra ver não o paciente com covid especificamente, mas o paciente que tinha outras comorbidades, ou muito idoso, ou que tinha muitas disfunções, ou que tinha neoplasia ou que já tinha outra coisa que contribuísse para isso. Mas o covid só, por si, o médico só veio ter maturidade de pedir cuidados paliativos para esse paciente especificamente, já da segunda onda para a terceira onda, quando o médico sabia como é que os pacientes evoluiriam. Eu acho que a questão que o paliativo trás é mais a questão de comunicação medial com a família. Porque muitas vezes o médico é um pouco longe da família e chama o paliativo. O paliativo vem junto e faz esse contato com a família. O médico acaba conhecendo mais a família, se integrando mais com a família. O médico acaba sabendo como conduzir um pouco mais, no sentido de evitar sofrimento, evitar dor desnecessária. O fato de a doença ser muito nova, o paliativo quando era só COVID, não tinha outra coisa, quando o médico chamava, era já muito tardio. E muitas vezes, meio que a contragosto. Não existia uma concordância plena de todo mundo. Vai paliar esse cara, tem 40 anos de idade? Vamos pedir pro paliativo para ver, vai limitar alguma terapia? O que que vai ser feito com esse paciente? A equipe do paliativo vai ter alguma limitação, deixar de investir? Existia muito temor de não deixar de fazer as coisas, porque a equipe da UTI também era uma equipe nova, não era uma equipe que trabalhava na UTI há muito tempo, isso na segunda onda. Acho que o médico chamou o paliativo muito pouco para os pacientes que eram COVID puramente. Quando o médico chamou o paliativo e era de plena concordância, era mais no aspecto de outras doenças de base. Eram pacientes idosos, ou que tinham grandes disfunções ou uma funcionalidade muito ruim. Nesses, foi como paliativo normal nas outras UTI. O médico, na verdade, só otimizou melhor a terapia, só muitas vezes definiu melhor, mais claramente, a questão da ida e saída da UTI. O médico viu realmente que trazia conforto e alívio, no sentido de deixar a família mais próxima. Liberava para família vir visitar também, mesmo sendo COVID, e trouxe essa possibilidade. A família pôde ter acesso mesmo na uti ao

paciente, com isolamento, com medidas. Quando chamava realmente, eu acho que melhorava e humanizava mais a assistência como um todo. Mas nesses pacientes que estavam com alguma disfunção ou outra, que tinha alguma coisa, doença de base antes. Quando era definido paliativo, a gente tinha um acesso maior à família. Eles entravam. A gente conseguia acabar dando alta mais fácil para a enfermaria, porque já tinha uma limitação terapêutica bem definida, já existia um acordo do que iria ser feito com esse paciente lá fora, em caso de piora. Não existia o temor de você mandar o paciente para a enfermaria e a enfermaria se desesperar com o paciente, se ele ia voltar precoce de novo ou não, se eles iam dar conta. Porque já tinha definido direito um plano terapêutico, até onde se iria com aquele paciente, o grau de cuidado que seria. A enfermaria acabava participando também dessa decisão. Então era mais clara a saída dos pacientes. Tanto a família tinha mais acesso, como o médico também ficava um pouco mais confortável, porque a família entrava junto, o médico conversava e ficava todo mundo aliviado, por estar conversando abertamente com o paciente, com a família. O médico conseguia dar alta do paciente mais claramente, porque agora existia a concordância em papel, em termo e inclusive participação da enfermaria também.

PARTICIPANTE MED 16

As ações de cuidado paliativo eu lembro demais. Lembro de um paciente aqui do hospital, de 28 anos, que tinha uma leucemia aguda e também covid e eu fiz uma extrema unção. Eu segurei o celular, filmando o paciente, e na tela estava o padre, a mãe, a irmã e o doente. Eu chorava tanto que eu segurava a mão pro celular não cair. A mãe perguntou se eu podia, e eu disse que sim. E nós médicos fizemos isso, para ficar mais perto da família, porque estava bem no começo mesmo, era maio de 2020, a primeira onda. Eu fiz. Ele tinha 28 anos e faleceu. Tive uma experiência também importante, uma amiga minha, médica, que estava gestante. Tive duas gestantes gravíssimas que saíram.

PARTICIPANTE TEC 17

As ações de cuidado paliativo que eu conseguiria enxergar seriam as seguintes. Primeiro é o contato mais precoce com a família, através do telefone. Ao mesmo tempo que havia uma dificuldade técnica, porque os familiares não tinham contato conosco, mas o recurso telefônico foi o necessário, foi o que pudemos fazer com os familiares. Presencial era um pouco mais difícil. Muitas vezes esse contato presencial acontecia quando o paciente já vinha a óbito, que o médico tinha que dar a notícia presencial, para o familiar. Mas um ponto foi essa questão da entrada, no método da limitação de cuidados do paciente por telefone, foi de maneira remota, que dependendo da maneira como o médico se posicione, não é um contato que precisa ser extremamente distante, ou seja, dá para a gente conseguir ter um mínimo de empatia com esse recurso telefônico, se bem utilizado. Dependendo muito, na verdade, da capacidade de comunicação do médico. Um outro ponto específico é que a equipe, infelizmente, não conseguiu ter a participação de maneira tão frequente quanto nós

gostaríamos da equipe de cuidados paliativos. Porque a demanda foi muito grande em outros setores também do hospital. Muitas vezes o médico, como equipe de uti, tinha que se encarregar totalmente dessa questão do diálogo com a família, empático. Houve um pouco mais de autonomia nesse sentido. O médico tinha como fazer algumas intervenções mais precocemente com os familiares, uma vez que o médico conseguia perceber a irreversibilidade do quadro clínico do paciente, da gravidade. O médico conseguiu, através do mesmo instrumento telefônico, fazer uma comunicação mais célere. Contato telefônico e a celeridade que o médico conseguia fazer dessa comunicação através do telefone. Principalmente mais com a família de fato. Muitos familiares chegaram para o médico nesse período do covid, e lamentando que não conseguiam estar presentes, que queriam ver os pacientes mesmo eles já estando em limitação terapêutica. Queriam vê-los partir, mas infelizmente o médico não podia permitir a entrada, por conta do risco de contaminação que era muito alto. O médico tentava ser o mais empático possível por telefone, e presencial quando realmente o paciente já vinha a óbito. Não poucas vezes a gente escutou de familiares que havia um sofrimento muito grande porque não conseguiram se despedir. Muitas vezes, infelizmente, não houve nem a oportunidade de que fosse realizado um velório, Muitas vezes o paciente tinha que sair da uti para o necrotério, do necrotério diretamente para o cemitério, sem passar pelo velório. A família não tinha nem o direito de velar o corpo. Foi uma, infelizmente, uma tônica no Brasil como um todo. O médico tinha até que já entrar um pouco nesse mérito dos cuidados paliativos post mortem, junto aos familiares, antes mesmo do paciente sair da uti, do corpo sair da uti. Porque a gente já sabia que era um corpo que não podia ser velado. Ao longo do tempo, o médico conseguiu uma flexibilização nesse sentido, mas no começo da pandemia foi algo muito desgastante, não somente para os profissionais de saúde mas também para os familiares. O médico procurou aplicar, não de rotina, como checklist, o spikes. Normalmente como o médico já tinha leitura prévia e experiência no dia a dia, o médico acabava aplicando o spikes, sem necessariamente estar ali com uma caneta e o papel na mão para ver se eu acertei o s, se eu acertei o p, se eu acertei o i. Mas o médico aplicou o que a gente sempre foi treinado a fazer. Abordar o paciente. Abordar a família. Algumas oportunidades o médico teve de explicar para o paciente que ele precisava ser entubado. Houve algumas oportunidade fazer isso também como uma forma de intervenção. Explicar que nós trataríamos da melhor forma possível do quadro clínico, até quando ele estava consciente. Os médicos tinham a oportunidade de dar conforto também nesse sentido, que todo o apoio da equipe da uti ele teria mesmo estando desacordado, sedado. Teve uma experiência lá na uti em que houve um dia em que a equipe estava totalmente desmotivada a trabalhar. A plantonista chegou para mim e disse que a equipe estava totalmente desmotivada porque a equipe estava perdendo muito paciente. Eu falei que entendia e que então iríamos parar para poder conversar um pouco. Então essa questão também dos cuidados paliativos, no sentido mais amplo de aliviar o sofrimento, também o médico tinha que fazer com a própria equipe. Tive que fazer algumas intervenções por 2 vezes pelo menos lá na uti, porque a equipe estava com dificuldade importante de aceitar também a gravidade dos pacientes. Foi um período muito duro para nós. A equipe tinha que conversar. Tinha que conversar sobre o morrer dos pacientes, a gravidade, a irreversibilidade, por mais que a equipe fizesse todos os esforços, a gente sabe que houve muita frustração por parte de muitos profissionais. A equipe teve que trabalhar essa parte de conforto, de cuidados paliativos, com a própria equipe da uti. Eu médico tive que fazer algumas intervenções com a própria equipe. Até nisso a equipe teve que se virar um

pouco, como equipe de uti. E saber cuidar, saber acolher, saber ouvir o medo, a dúvida, o receio, a angústia, e motivar também a equipe para continuar o trabalho. Os próprios colegas.

PARTICIPANTE PSICO 18

As ações de cuidado paliativo da psicologia eram mais casos pontuais. A psicologia atendia individualmente e comunicava as percepções com a equipe da uti. Não era tanto um diálogo com a equipe de cuidados paliativos. Nessa época, o trabalho da psicologia estava sendo muito de maneira online. Eu atendia às solicitações da equipe e as interconsultas, não fazia busca ativa nesse momento. Hoje em dia eu faço a busca ativa. Nessa época da pandemia, ficou mais por solicitação da equipe. A psicologia ia atender o paciente e fazia muito esse movimento de aproximação afetiva do paciente com a família. Através de videochamada. Quando, por exemplo, o paciente não estava consciente, quando eu fazia um atendimento à família online, porque nesse momento eu estava fazendo atendimento online à família. Às vezes eu colocava a possibilidade de envio de mensagens de áudio para os pacientes, mesmo eles não estando conscientes. A psicologia colocava essa possibilidade. A família enviava para o aparelho da uti uma mensagem de áudio. Podia ser uma música também. Algo que fizesse parte da história do paciente, da família, que fizesse sentido para a família. E aí eu colocava para o paciente, para reproduzir para o paciente poder escutar, ao ouvido do paciente. E quando o paciente estava consciente, orientado, a psicologia colocava a possibilidade da videochamada e fazia a videochamada com a família, como forma de aproximar a família do paciente, de alguma maneira. Para promover essa aproximação afetiva com a família. Para dar um conforto tanto ao paciente, como à família nesse momento. Uma estratégia também de conforto emocional. E tem o atendimento online também, individual, à família. Eu também ofertava, eu telefonava para os familiares, para os contatos que tinha na anamnese do serviço social. Eu telefonava para os familiares ofertando essa possibilidade de cuidado. Alguns eram receptivos a essa oferta de atendimento psicológico e alguns não. Quando eles eram receptivos, eu agendava um horário, de atendimento no ambulatório. Eu telefonava. Era por chamada de vídeo do whatsapp mesmo, telefonava pelo whatsapp. Era um atendimento semanal.

PARTICIPANTE TEC 19

As ações de cuidado paliativo eram assim, os técnicos de enfermagem faziam tudo. Mesmo um paciente sendo paliativo. Às vezes os técnicos de enfermagem até se estressavam. A equipe técnica se estressava porque um paciente paliativo tinha prescrição de tudo. Tinha prescrição de clister. Porque a questão da paliação na nossa cabeça é medida de conforto para um paciente. Que se resumiria em ofertar oxigênio, para que esse paciente não viesse a sofrer por falta de ar, principalmente no covid e a questão da sedação, de não estar sentindo dor. E as prescrições tinham tudo, elas tinham tudo. Ela tem mudança de decúbito, de 2 em 2 horas, ela tem clister se o paciente não tá evacuado. Os pacientes com todas as drogas. Sedoanalgesia, bloqueados, com droga vasoativa. As drogas vasoativas aumentavam de vazão, e às vezes os técnicos de enfermagem apostavam que iriam fazer tudo e podia ser que o paciente até melhorasse, mas raramente via isso. Algumas vezes a equipe chegou a presenciar pacientes

muito graves, com câncer, que já vinham de um câncer paliado, e pegava covid. E a equipe viu o paciente sair de alta, sair bem, ir para a enfermaria e se recuperar para ir pra casa. E outros que nem eram tão graves, pacientes jovens, que de repente, descompensava tanto que entrava para paliação. E todas as medidas e cuidados de enfermagem eram os mesmos, mesmo se fosse paliativo e se não fosse. Não mudava. Na prática não muda muito. Continuava tudo, tudo, tudo, tudo era feito.

PARTICIPANTE TEC 20

As ações de cuidado paliativo são de priorizar mais sedação, mais analgesia, outros cuidados, além dos cuidados que a enfermagem já tem, que é em questão da mobilização daqueles que estão muito graves. Mas priorizar analgesia, priorizar o conforto do paciente. Embora a equipe saiba o desfecho e a equipe espere um desfecho. Pacientes desconfortáveis respiratoriamente, ajustar parâmetros de ventilação, melhorar analgesia, melhorar sedação, priorizar conforto, no geral. É isso o que a enfermagem já tem em mente. Para esses pacientes não houve diferença. A equipe sabia o diagnóstico, sabia tudo, mas a prioridade do conforto para esses que chegavam ao fim, a qeuipe dava atenção maior. Já que já existiu falha terapêutica, é dar o conforto para o paciente. A enfermagem, o que é que a gente faz com esses pacientes. Tenta melhorar a mobilização, com as mudanças de decúbito, hidratação de pele. Conforto para que o paciente fique o mais confortável possível, tanto posicionado, como a questão da higiene, do banho, da higiene íntima no leito, é isso que a enfermagem gente faz. Outros pacientes também muito graves, como a enfermagem não conseguia mobilizar da forma correta, que é com a mudança de 2 em 2 horas, de mudar e lateralizar, a enfermagem faz uma mudança que a chama de semidecúbito, que coloca os coxins para descomprimir a pelve, descomprimir os trocânteres e as regiões que encostam nos colchões e intensificar a hidratação da pele para que não aconteça a abertura de úlcera. O nosso índice de úlcera que acontece aqui na unidade é bem baixo, por conta disso. Porque a enfermagem tem essa priorização de cuidados de pele, de mudança de decúbito e de conforto ao paciente também. Eu acredito que em relação aos cuidados de enfermagem não muda. Quando a unidade fechou para covid e foi só covid, os cuidados permaneceram os mesmos, embora a equipe estivesse toda paramentada, mas a enfermagem tentou fazer o máximo para manter o mesmo padrão de cuidados de enfermagem com o paciente. Os banhos, as higienes e tudo.

PARTICIPANTE TEC 21

As ações de cuidado paliativo eram amenizar a dor, amenizar a dor. Ter mais próximo os membros queridos. Proporcionar o conforto. A equipe diminui o que traz mais transtornos para ele. Eu acho que o que a equipe puder minimizar para eles, é melhor. Tanto para eles como para a família. Porque é um momento bem delicado a família aceitar que o paciente, que aquele ente querido ele está morrendo daqui a uns dias. É bem difícil. Porque os familiares não tiveram esse contato, não pôde ter esse contato, por conta da contaminação. Não puderam vivenciar esse momento. Porque foi muito rápido. Não lembro desses pacientes, se teve melhora, para ir para a enfermaria. Foi a óbito.

PARTICIPANTE TEC 22

As ações de cuidado paliativo? Não teve, não. Aqui demora muito para entrar em processo de paliativo. Não lembro de paliativo lá não. Porque tem até a equipe de cuidado paliativo e não lembro de eles terem ido lá. Não teve não.

PARTICIPANTE TEC 23

As ações de cuidado paliativo tinha essa lacuna, de explicar para a equipe o que é que se tinha de fazer, mas o básico do cuidado paliativo a equipe sabia, que tinha que dar o melhor para o paciente. O conforto, evitar o sofrimento. Evitar procedimentos invasivos que não vão trazer benefício ao paciente e isso a equipe aceitava. Sem ter muita indagação. Se o paciente fosse consciente ou traqueostomizado, geralmente os médicos colocam em bomba de morfina para o paciente não ficar em desconforto respiratório. Muitas vezes a equipe mantinha o paciente sedado, porque ele não conseguia sair da ventilação mecânica, mas é basicamente isso. Fazer todos os procedimentos devidos, os cuidados da enfermagem, e caso precise, alguma medicação que seja para sintomático. Chega um ponto que a equipe médica mesmo decide tirar até a glicemia, tirar tudo que é de invasivo, e fazer uma punção hipodermóclise para o paciente, para ficar uma bomba de morfina em dosagem baixa. Mas muitas vezes, a enfermagem em si aceita quando chega uma equipe e diz que o paciente não tem muito o que fazer, e aí a gente vai adequando o cuidado na medida do possível.

PARTICIPANTE TEC 24

As ações de cuidado paliativo era o uso das bombas de morfina, eram os cuidados. Pronação, para tentar aliviar sensação de sufoco, sensação de falta de ar. Era a questão do conforto de feridas. A equipe teve muito acessório para elevar o calcâneo, elevar a cabeça, fazer a mudança de decúbito. Era o conforto, no uso de medicação e o conforto mesmo na forma de prontuário afetivo. A equipe colocava que era católico, a equipe colocava música. Teve pacientes que ficavam ouvindo música o dia todo. Teve paciente que ele ficava pedindo as músicas. Pacientes que eram católicos, teve o terço que a família trouxe, teve fotos. Essa questão da audição, de dizer, de repassar, teve bastante esses cuidados, dentro do cuidado paliativo. Quando é trabalhado o prontuário afetivo ele gera uma afetividade. Ele gera uma transferência de relação paciente e equipe, porque você conhece como é o apelido carinhoso que a família trata, você conhece o quê que ele é, aí às vezes o prontuário afetivo vem e mostra que ele é pai de 4 filhos. Você começa a ver essa transferência. É bastante interessante essa transferência. A equipe chama pelo apelido carinhoso, vê qual a música que ele gosta. Tinha um paciente que gostava de Roberto Carlos, então a equipe, mesmo ele entubado, colocava a música do Roberto Carlos porque sabia que ele estava ouvindo, de qualquer forma, aquela canção do Roberto Carlos. Músicas evangélicas, músicas católicas. Tinha um que gostava de brega. A equipe tinha essa parte bem dinâmica. Na primeira onda a equipe teve aquele pavor, pavor mesmo, de morte. Porque a equipe tinha a sensação de que quando vinha

pro leito covid, estava sendo praticamente sentenciado à morte. A equipe vestia a roupa, avental, luva, outra luva por cima, um óculos, uma máscara, o gorro, aquela sensação de sufoco, calor, até pela questão mesmo da adrenalina de estar lidando com uma doença que a equipe não conhecia. E a equipe passava 6 horas no leito. O técnico não podia sair. O técnico só saia quando havia a troca de equipe. O técnico ficou 6 horas aqui dentro desse leito, fazendo banho e olhando para o paciente. E aquela sensação de medo. A primeira onda foi. Na segunda onda não, como já havia os mecanismos, já havia a questão do elmo, a questão da pronação, que melhorou bastante. Esses mecanismos, quais as medicações davam para serem trabalhadas. A equipe não deixou de ter os cuidados, mas respirou mais aliviado, em certos momentos. Já tinha a vacina também. A primeira dose, a segunda dose já estava sendo aplicada. Não dava para ter aquele cuidado paliativo na primeira onda. Era aquele cuidado, mas a gente cuidando ali para salvar, não aquele cuidado de conforto. Era aquele cuidado mesmo do vamos, vai dar certo, vai sair dessa, e vamos, vai ter saúde. Eu sou terapeuta holístico também. Fazia a auriculoterapia, acupuntura, alguma terapia para ansiedade. Fazia o Reiki, fazia aquela questão do cuidado. Vinham as técnicas que também eram evangélicas e faziam oração. Fazia quem era católico, ficava rezando do lado ali. Houve esse lado de fé também, esse lado de fé e o lado terapêutico. A equipe fazia bem isso. Do caso das músicas, colocava ali as músicas. Nós tivemos apresentação de violino. Reflexologia podal, a questão do Reiki, a questão da auriculoterapia dava para fazer. A acupuntura com agulha, com alguma massagem auricular.

PARTICIPANTE TEC 25

As ações de cuidado paliativo, na minha parte o que iria diferenciar para mim? praticamente nada. Porque o que é o meu? é medicação, que eu faço o que estiver prescrito e o conforto. Eu não vou deixar de banhar, eu não vou deixar de fazer a mudança de decúbito, eu não vou deixar de fazer higiene oral. Os meus cuidados, eles não vão deixar de serem prestados porque o paciente vai morrer, a não ser que tenha na prescrição, não fazer mais glicemia, não fazer mais isso. Mas o conforto, que é a mudança de decúbito, que é a higiene oral, essas coisas a enfermagem nunca deixou de fazer. Nossa parte a enfermagem faz. Na minha percepção, o cuidado de enfermagem continua o que é para o conforto do paciente. Porque eu vejo os médicos retirarem o que vai acrescentar sofrimento para o paciente. O conforto de uma verificação de pressão? porque tem o conforto do paciente e do familiar. Uma glicemia, vou furar os pacientes de 6 em 6 horas? porque muitas vezes também nem vai mais comer. Estou dizendo no paliativo. Os médicos vão tirando as coisas deles da prescrição. Mas o nosso cuidado do conforto é tudo o que vai favorecer o paciente. Eu sei que vai ter hora que a pele dele não vai mais responder, mas eu não vou deixar de fazer minha parte de cuidado, de banhar. O nosso cuidado de enfermagem em relação ao conforto, que é banho, que é higiene, que é mudança de decúbito, se ele estiver suportando, vai continuar. Muda nada da enfermagem não. O que vai diminuir é o que estiver na prescrição a menos, que a enfermagem não vai fazer.

PARTICIPANTE TEC 26

As ações de cuidado paliativo eram a higienização, mudança de decúbito, questão de sedação, morfina para que eles não viessem a sentir dor. Conforto respiratório também. Porque o covid ele afetava mais a parte respiratória. Na forma de medicação muda sim, porque quando o paciente é paliado, muita coisa zera, não tem glicemia, só tem uma hidratação. A alimentação, e a medicação para aliviar a dor. Dar o conforto só.

PARTICIPANTE TEC 27

As ações de cuidado paliativo eram comunicadas à equipe. Os médicos comunicavam que aquele paciente não tinha prognóstico e não aumentava mais a noradrenalina, não alterava mais medicação. Às vezes não dialisava mais. Não conseguia mais mudar um decúbito. Às vezes a enfermagem nem conseguia mais banhar, fazia só uma higiene. E, na época, sugeriam umas espumas, que aí a enfermagem fazia só uma higiene, sem mexer no paciente. Esses cuidados. Só que não tinha aquela presença da família como antes. O cuidado normal de enfermagem. É o mesmo. Se pudesse mudar decúbito, a enfermagem mudava. Se tivesse condições de alimentar, alimentava. Continuavam os mesmos cuidados. A higiene oral. Todos os cuidados de enfermagem mantinha, mesmo sabendo que ele era paliativo. Porque houve casos que os pacientes melhoraram e saíram da paliação. Talvez o cuidado de enfermagem, o cuidado da medicina, o próprio organismo dele e Deus, que às vezes não quer que a pessoa vá naquele momento. Alguns, não muito, mas tiveram casos. E o cuidado de enfermagem fez com que o paciente melhorasse de uma escara. Mudança de decúbito, o banho que às vezes era diferenciado, era um banho mais rápido, ou uma higiene. Os cuidados eram os mesmos de todos pacientes, só que às vezes a enfermagem tinha que ter um pouco mais de cuidado para que ele não se agravasse e não antecipasse a morte dele. Paciente marcante foi uma paciente que era paliado e saiu da paliação. Para mim foi uma coisa que foi decidido como paliado, que não tinha mais condições e o paciente mesmo teve uma melhora. Conseguiu sair da UTI junto com a família. Não sei se ele teve alta hospitalar, mas ele foi paliado, mas saiu da paliação e voltou. Saiu da intubação, teve uma resposta. Foi um caso que para mim foi importante. Eu acho que é uma área que a gente tem que melhorar mais, porque às vezes acontece que a enfermagem é comunicado que o paciente é paliativo, e na hora que acontece o óbito na uti, é bem mais difícil para a família estar presente. Ou então mesmo em uma visita, quando o paciente morre. Como já aconteceu, a equipe chamou para rezar, para conversar com a pessoa, mesmo sabendo que às vezes está sedado, mas para dar um conforto para a família. É um ente querido daquela pessoa. No caso do covid a equipe não teve esse acesso ao familiar, para ele vir no momento terminal daquela pessoa. Ele não teve aquele acesso. Mesmo possuindo o celular, mas aquela pessoa não podia falar. Às vezes o expressar daquele familiar ajuda muito no momento até da partida daquele paciente, para ele ir em paz.

PARTICIPANTE TEC 28

As ações de cuidado paliativo é você dar conforto ao paciente. Você alivia a dor, dá conforto em palavras, conforto emocional também, o conforto psicológico. Tenta dar para ele qualidade de vida, o máximo possível naquele momento de dor e nova situação. Que o paliativo não é uma questão de não ter uma expectativa de vida. Pode sim ter uma qualidade de vida, mesmo com diagnóstico de paliação. Na assistência da Enfermagem, quando estava na UTI, a equipe

fazia videochamada com a família, já que eles não poderiam entrar em contato com o paciente no momento. Pra aproximar e dar esse conforto ao paciente também, da parte afetiva. Fazia massagens. Utilizava o Reiki, fazia a oração. Isso através também de massagem também, que é o toque das mãos. Eu dava muito o toque das mãos. Os pacientes mais debilitados emocionalmente, depressivos ou até mesmo com ansiedade, eu fazia muito o Reiki, aplicava o Reiki neles, onde eles tinham uma resposta boa. Em termos clínicos e em termos emocionais. A equipe fazia essa aproximação com brincadeiras, perguntava do que eles gostavam, botava música do cantor que eles gostavam. Alívio da dor através de medicação ou uma posição que ele gostasse de ficar e vídeos. A equipe, sempre que podia, pegava um celular ou computador na unidade e colocava vídeo para eles assistirem, pra ter esse momento de vida comum, normal, como de qualquer outra pessoa.

PARTICIPANTE TEC 29

As ações de cuidado paliativo eram basicamente a mesma coisa. Medidas de conforto. Mudança de decúbito para evitar úlceras de pressão ou extensão das úlceras de pressão. Medicações para dor. Massagem. Um posicionamento melhor no leito.

PARTICIPANTE TEC 30

As ações de cuidado paliativo, o que eu via eram algumas condutas que eram diferentes, de médico para médico. Alguns enxugavam a prescrição quando estava exclusivo, para proporcionar um melhor conforto. Retirava mensuração de pressão arterial, verificação de glicemia. Mas outros médicos achavam que era importante, aí mudavam. Mas era basicamente isso, proporcionar conforto. E muitos médicos enxugavam também a questão de antibióticos. Os que estavam em limitação terapêutica não. Eu via que continuava. Mas os exclusivos os médicos retiravam tudo, era mais conforto mesmo. A questão da família é que era mais difícil, era tudo muito restrito. Era bem difícil a equipe conseguir fazer com que a família entrasse, acompanhasse mais de perto. Os cuidados continuavam basicamente. O conforto, a questão do conforto, eram muito parecidos com os outros que ainda não tinham limitação terapêutica. Eram medidas de conforto. Continuava a mudança de decúbito, quando o paciente tolerava. Continuavam os banhos da mesma forma. Falando do cuidado em si. Continuavam os medicamentos sintomáticos. Todos continuavam da mesma forma. Da mesma forma que para os outros a enfermagem proporcionava. A mudança de decúbito, elevação dos membros. Tudo a enfermagem fazia da mesma forma para eles. Não é porque era paliativo que a enfermagem deixava de mobilizar. A não ser os pacientes que apresentavam instabilidade hemodinâmica. Aí a enfermagem deixava mais quieto, não mexia. Mas os que podiam a enfermagem fazia todas as medidas. Mudança de decúbito, febre, fazia antitérmico. Fazia todos os sintomáticos, todos eram feitos.

PARTICIPANTE TEC 31

As ações de cuidado paliativo que consigo lembrar eram o conforto. Por mais que seja um paciente paliado, tem que fazer mudança de decúbito, higienização, muito importante. Banho no leito, medicação analgésica para dar o conforto. Não é muito diferenciado de um paciente que não é paliativo. A enfermagem dá todos os cuidados, toda a atenção. O COVID, o que eu percebi nos pacientes paliativos, é que a enfermagem não tinha muito a rotina de fazer mudança de decúbito, até pela doença em si, porque às vezes tinha paciente que não suportava decúbito. E pelas suas condições em geral, às vezes eles ficavam hipotensos. Permaneciam mais tempo todo em decúbito dorsal. Não tinha muita mudança de decúbito, até pelo seu estado, pela instabilidade hemodinâmica. Era diferenciado só isso mesmo, que eu via diferenciado dos pacientes clínicos da UTI para os de COVID. Porque eles eram muito instáveis hemodinamicamente por conta da respiração. E a maioria dos cuidados mesmo era só higiene, mudança de decúbito, às vezes, quando dava e a parte da sedação e analgesia. Não tinha muita diferença não de um paciente clínico. A não ser mesmo o risco de contaminação. E eu percebia também que os pacientes com COVID, a equipe evitava ao máximo fazer aerossóis, ficar desconectando o tubo dele. No manuseio, esses cuidados que tem que ser dobrados, que é diferente dos pacientes clínicos.
