# Supplementary material for: Palliative Care for SARS-CoV-2 Patients in the Intensive Care Unit: A Comprehensive Study
Source: Rev Bras Enferm. 2024 Jun 28;77(Suppl 1):e20230218. doi: 10.1590/0034-7167-2023-0218 (PMC11213540; doi:10.1590/0034-7167-2023-0218)
Supplement: Supplementary file 2 [file 0034-7167-reben-77-s1-e20230218-suppl2.pdf]

README

Este guia refere-se ao banco de dados do artigo: **Cuidados Paliativos ao paciente com SARS-COV-2 em unidade de terapia intensiva: estudo compreensivo.**

Pesquisador correspondente: Marilia Alves Furtado. Universidade Estadual do Ceará. Email: marilia.furtado@aluno.uece.br

Data de coleta dos dados: Os dados foram coletados durante os meses de junho a julho de 2022.

Visão geral dos dados e arquivos: O arquivo de dados é composto por uma parta qualitativa, referente à transcrição dos aúdios gravados durante a realização das entrevistas com os participantes do estudo e por uma parte quantitativa, através de tabela com os dados socioepidemiológicos dos participantes.

Descrição dos métodos de coleta ou geração dos dados: Os dados foram coletados por meio de entrevista, realizadas de modo individual com cada participante da pesquisa. Os participantes foram convidados a discorrer sobre o seguinte questionamento: Como você descreve as diferentes ações de cuidados paliativos fornecidos aos pacientes com SARS-COV-2 internados em sua unidade de trabalho? A pergunta referia-se às ações paliativistas realizadas durante o período pandêmico, em unidades de terapia intensiva. Além disso, os participantes responderam questionamentos sobre suas caracterísitcas sóciodemográficas.

Descrição dos métodos usados para o processamento dos dados: Os dados qualitativos foram analisados a partir do referencial teórico escolhido, com a organização dos dados sendo auxiliada pelo software de análise qualitativa IRAMUTEQ. Os dados tabulares foram descritos em termos de frequência e média, não sendo necessário utilizar software de análise estatística.

Informações específicas dos dados: A fim de manter-se o anonimado dos participantes, os dados qualiativos e tabulares estão descritos de acordo com a categoria profissional dos participantes, sendo ENF referente ao profissional enfermeiro; Técnico referente ao técnico de enfermagem; MED referente ao profissional médico; FISIO referente ao profissionao fisioterapeuta; e PSICO referente ao psicólogo.

Em relação aos dados tabulares, este possui a seguinte legenda:

Identificação segundo a legenda descrita acima para os dados qualitativos.

1. Idade, em anos:

2. Sexo: Feminino (1) Masculino (2)

3. Profissão:______________________

4. Escolaridade: Nível técnico (1) Nível Superior (2) Especialização (3) Mestrado (4) Doutorado (5) Pós-doutorado (6)

Se pós-graduação, especificar: ___________________________

6. Você teve alguma cadeira/curso de Cuidados Paliativos durante sua formação acadêmica e profissional? Sim (1) Não (2)

7. Especialização em Cuidados Paliativos? Sim (1) Não (2)

8. Trabalhava em Uti anteriormente? Sim (1) Não (2)

9. Quanto tempo de atuação profissional? (tempo em anos) __________________

10. Tempo total de atuação em UTI (em anos) _________

11. Em caso de experiência em Cuidados Paliativos, tempo total em anos; ____________
