# Supplementary material for: Palliative Care for SARS-CoV-2 Patients in the Intensive Care Unit: A Comprehensive Study
Source: Rev Bras Enferm. 2024 Jun 28;77(Suppl 1):e20230218. doi: 10.1590/0034-7167-2023-0218 (PMC11213540; doi:10.1590/0034-7167-2023-0218)
Supplement: Supplementary file 3 [file 0034-7167-reben-77-s1-e20230218-suppl3.pdf]

IDENTIFICA€ÇO;IDADE (ANOS);SEXO (1
ENF;44;1;Enfermeira;2;;2;2;2;6;1;6
FISIO;42;1;Fisioterapeuta;4;Fisioterapia hospitalar
PSICO;34;1;Psicologa;4;Preceptoria em Saude
TECNICA;31;1;T,cnica;2;;1;2;1;11;4;5
ENF;34;1;ENFERMEIRA;4;CARDIOVASCULAR;2;2;1;12;5;5
T⮈CNICA;37;1;T⮈CNICA;2;;1;2;1;18;16;8
ENF;34;1;ENFERMEIRA;5;UTI;2;2;1;12;10;5
T⮈CNICA;40;1;TECNICA;2;;2;2;1;13;5;5
TECNICA;33;1;TECNICA;1;SAUDE DO IDOSO;2;2;1;14;6;6
TECNICO;42;2;TECNICO;2;URGENCIA E EMERGENCIA;1;2;1;18;1;8
MEDICO;53;2;MEDICO;4;CLÖNICA E CUIDADOS PALIATIVOS;1;1;1;27;20;10
ENF;40;1;ENFERMEIRA;3;PRECEPTORIA EM SAéDE;2;2;2;10;1;7
ENF;34;1;ENFERMEIRA;4;UTI;1;2;1;10;4;5
TECNICO;40;2;TECNICO;3;TERAPIAS HOLÖSTICAS E NUTRI€ÇO CLÖNICA E HOSPITALAR;1;2;1;14;9;7
TECNICA;41;1;TECNICA;1;ENFERMAGEM DO TRABALHO;2;2;1;18;10;
TECNICA;54;1;TECNICA;2;;1;2;1;20;20;14
FISIO;32;1;Fisioterapeuta;3;CARDIORESPIRATàRIO E FISIOTERAPIA HOSPITALAR;1;2;1;12;5;
MEDICO;42;2;MEDICO;3;CLÖNICA E UTI;2;2;1;17;10;5
TECNICA;40;1;T⮈CNICA;2;;1;2;1;20;20;
ENF;36;2;ENFERMEIRO;5;UTI/NEFRO E DERMATO;2;2;1;14;12;
MEDICA;43;1;MEDICA;3;CLINICA MEDICA E PRECEPTORIA M⮈DICA;2;2;1;12;8;3
MEDICO;39;2;MEDICO;3;CLINICA MEDICA E UTI;1;2;1;14;3;4
TECNICA;46;1;TECNICA;2;;2;2;1;27;7;
TECNICA;29;1;TECNICA;2;;2;2;1;10;3;
FISIO;44;1;Fisioterapeuta;3;CARDIORRESPIRATàRIA/FISIO HOSPITALAR E PRECEPTORIA EM SAUDE;2;2;1;20;10;5
ENF;36;1;ENFERMEIRA;3;UTI E AUDITORIA;2;2;2;13;1;4
FISIO;48;1;Fisioterapeuta;4;CARDIORRESPIRATàRIA/PRECEPTORIA EM SAUDE/PSICOMOTRICIDADE E RESPIRAT
ENF;35;1;ENFERMEIRA;4;UTI;2;2;1;12;12;
TECNICA;32;1;T⮈CNICA;2;;1;2;1;8;8;6
TECNICA;44;1;TECNICA;1;;2;2;1;15;14;7
ENF;42;1;ENFERMEIRA;4;UTI/CP/TEOLOGIA DA EDUCA€ÇO/UE/MATERNO-INFANTIL-GO/TECNOLOGIAS INOVATI

2);PROFISSÇO;ESCOLARIDADE (1 2 3 4 5 6);SE PàS

Cardiovascular e preceptoria;2;2;1;19;19;19
Terapia cognitivo comportamental e Oncohematologia (resi);2;2;1;10;2;2

QUAL?;DISCIPLINA/CURSO DE CP NA FORMA€ÇO? (1

2);Especializa‡Æo em CP;UTI ANTERIORMENTE? (1

2);TEMPO DE ATUA€ÇO;TEMPO DE UTI;SE TEMPO DE CP
