# Supplementary material for: Palliative Care for SARS-CoV-2 Patients in the Intensive Care Unit: A Comprehensive Study
Source: Rev Bras Enferm. 2024 Jun 28;77(Suppl 1):e20230218. doi: 10.1590/0034-7167-2023-0218 (PMC11213540; doi:10.1590/0034-7167-2023-0218)
Supplement: Translation

# Translation: Cuidados Paliativos ao paciente com *SARS-CoV-2* em unidade de terapia intensiva: estudo compreensivo

**DOI:** 10.1590/0034-7167-2023-0218pt

**Authors:** Marilia Alves Furtado, Vitória Pessoa Nogueira, Maria Clara Passos Araújo, Virna Ribeiro Feitosa Cestari, Vera Lúcia Mendes de Paula Pessoa

**Affiliations:** I Universidade Estadual do Cearpa. Fortaleza, Ceará, Brasil

**Keywords:** Assistência Terminal, Covid-19, Cuidados Paliativos, Sars-Cov-2, Unidades de Terapia Intensiva

## Abstract

**Objetivo:**

Compreender as ações multiprofissionais quanto à paliação de pacientes em Unidade de Terapia Intensiva, acometidos pelo *SARS-CoV-2*.

**Métodos:**

Estudo compreensivo, qualitativo, realizado com 31 profissionais de Unidades de Terapia Intensiva de um hospital universitário, com base na Teoria do Fim de Vida Pacífico.

**Resultados:**

A análise dos discursos conduziu à identificação de duas categorias: “Ações multidisciplinares para promover o conforto no fim da vida” e “Cuidado paliativo durante o período pandêmico e o estímulo ao conforto emocional e espiritual”.

**Considerações Finais:**

Tornou-se evidente a necessidade de a administração local investir em medidas que minimizem as barreiras à implementação da assistência paliativa durante momentos de crise. A compreensão dos discursos ressaltou que profissionais não especializados podem fornecer cuidados paliativos básicos de maneira adequada, sem desmerecer a importância e a necessidade da presença de especialistas em Cuidados Paliativos em diferentes áreas hospitalares.

## INTRODUÇÃO

O Cuidado Paliativo (CP) é uma abordagem voltada para o aumento da qualidade de vida de pacientes e familiares que enfrentam problemas relacionados a doenças que ameaçam a vida, ou intenso sofrimento relacionado às questões de saúde, visando proporcionar suporte físico, psicossocial e espiritual^(1)^. Devido à natureza desse cuidado, a Organização Mundial da Saúde (OMS) considera o seu fornecimento essencial em situações de catástrofes e crises humanitárias, como a pandemia de *SARS-CoV-2*. Apesar de a maioria dos esforços em tais situações consistir em salvar vidas, medidas de alívio de sofrimento devem ser consideradas, proporcionando um cuidado de saúde embasado em princípios éticos^(2)^.

No contexto da recente pandemia de *SARS-CoV-2*, a restrição do número de profissionais em unidades fechadas e a necessidade de economia no uso de equipamentos de proteção individual (EPI) foram fatores limitantes para a presença efetiva de profissionais especialistas em CP em unidades fechadas, como a Unidade de Terapia Intensiva (UTI), levando à necessidade de que esse cuidado fosse fornecido, em grande parte, pelos profissionais assistenciais das unidades^(3)^.

A organização dos serviços de saúde para a adequação dos CP como eixo transversal, especialmente em cenários pandêmicos e de catástrofes, é essencial para assegurar um cuidado embasado nos princípios éticos, de conforto e qualidade. Considera-se que os princípios dos CP contribuem para o fornecimento de um cuidado de saúde integral, porém sua implementação em situações de crise e abalo do sistema de saúde torna-se desafiadora^(4)^.

Um estudo de revisão de escopo realizado com o intuito de analisar as evidências científicas sobre a inserção dos CP durante a pandemia de *SARS-CoV-2* não encontrou pesquisas realizadas no cenário da América do Sul^(4)^. Uma pesquisa realizada no Reino Unido, por sua vez, demonstrou o aumento exponencial do número de pacientes graves com *SARS-CoV-2*. O número de pacientes referenciados à equipe de CP teve um aumento considerável, passando de dois casos por semana a 51 casos semanais^(5)^.

Dessa forma, potencializar as escolhas assistenciais é fundamental. Apesar das perdas em tempos pandêmicos serem inestimáveis, torna-se urgente repensar a forma de enfrentar tais desafios em momentos futuros^(6)^. Destaca-se, portanto, a relevância de rever a prática de paliação fornecida por profissionais intensivistas durante o período pandêmico, em favor da reflexão acerca da provisão de tais cuidados em um cenário de limitada atuação de equipes especialistas, possibilitando embasamento para repensar estratégias futuras de enfrentamento.

### Referencial teórico-metodológico

A fim de possibilitar a presente reflexão, considera-se essencial a utilização de um arcabouço teórico que sustente a prática paliativista. A Teoria do Fim de Vida Pacífico (TFVP) apresenta-se como um valioso instrumento, devido à sua visível articulação com os princípios paliativistas, bem como à natureza prática de seus pressupostos, sendo capaz de guiar a atenção dos profissionais para uma assistência paliativa eficaz^(7)^.

As teóricas elencam cinco pressupostos para o alcance de uma morte pacífica, sendo eles: 1) ausência de dor; 2) experiência de conforto; 3) dignidade e respeito; 4) proximidade com pessoas importantes e 5) estar em paz. A TFVP sustenta a provisão de uma assistência em saúde que promova o bem-estar do paciente e da família de forma multidimensional, considerando a complexidade do ser na fase final do ciclo de vida^(8)^.

## OBJETIVO

Compreender as ações multiprofissionais quanto à paliação de pacientes em Unidade de Terapia Intensiva, acometidos pelo *SARS-CoV-2*.

## MÉTODOS

### Aspectos éticos

Os aspectos éticos da pesquisa com seres humanos foram respeitados, conforme a Resolução 466 de 2012 do Conselho Nacional de Saúde^(9)^. O estudo foi conduzido de acordo com as diretrizes de ética nacionais e internacionais e aprovado pelo Comitê de Ética em Pesquisa da Universidade Estadual do Ceará, cujo parecer está anexado à presente submissão. O consentimento livre e esclarecido foi obtido de todos os indivíduos envolvidos no estudo, por meio escrito.

### Tipo de estudo

Estudo compreensivo, de abordagem qualitativa, orientado pela ferramenta COREQ^(10)^. Estudos compreensivos focam na subjetividade da vida social, com o objetivo de compreender e interpretar a realidade dos fenômenos humanos gerados na sociedade^(11)^.

### Procedimentos metodológicos

####  Hipóteses 

Considerou-se como hipótese que profissionais não especialistas precisaram conduzir a paliação dos pacientes das unidades e que o cuidado paliativo foi fornecido por esses indivíduos.

####  Cenário do estudo 

O estudo foi realizado em um hospital universitário de grande porte, localizado no município de Fortaleza, Ceará, durante os meses de junho e julho de 2022. O cenário compreendeu as duas UTIs destinadas ao atendimento de pacientes graves acometidos pelo SARS-CoV-2. Cada uma das unidades contava com oito leitos de internação, totalizando 16 leitos para o atendimento ao paciente crítico com SARS-CoV-2.

### Fonte de dados

Os participantes da pesquisa foram os integrantes da equipe multiprofissional que atuavam nas referidas unidades do hospital durante a pandemia de SARS-CoV-2. Foram excluídos os profissionais que estavam ausentes de suas atividades por um período superior a 30 dias no momento da coleta, bem como aqueles admitidos por seleção específica para o atendimento da situação de pandemia por tempo determinado e que tiveram seus contratos vencidos. O levantamento dos sujeitos foi feito por meio da busca pela lista de profissionais atuantes nas referidas unidades no período pandêmico, obtendo tais dados junto às coordenações das equipes nas respectivas unidades.

Os participantes foram convidados via e-mail, contendo informações sobre a pesquisa, com a posterior assinatura do Termo de Consentimento Livre e Esclarecido (TCLE) de forma presencial, em caso de aceitação. A busca inicial pelos sujeitos resultou em uma população de 80 profissionais. Desses, três estavam de férias ou em licença, e um relatou não ter trabalhado nos setores durante o período da pandemia, resultando na exclusão de quatro sujeitos.

Além disso, 28 profissionais recusaram a participação, e três aceitaram participar, mas não compareceram no horário marcado e nem responderam novamente às tentativas de contato. Dos 43 profissionais restantes, seguiu-se a inclusão sucessiva de sujeitos até a saturação dos dados, com o encerramento das entrevistas após a coleta com 31 profissionais. Quanto à caracterização dos participantes, nove eram enfermeiros, quatro eram fisioterapeutas, quatro eram médicos, uma era psicóloga e 13 eram técnicos de enfermagem.

### Coleta e organização dos dados

A obtenção dos discursos ocorreu de forma individual, na modalidade virtual, por meio do aplicativo de reuniões virtuais *Google Meet*, em data e horário previamente indicados pelo participante. Foram realizadas entrevistas semiestruturadas, conduzidas pela pesquisadora principal do projeto, que possui título de especialista em Enfermagem em Terapia Intensiva e experiência em pesquisas de abordagem qualitativa. A coleta foi interrompida após a observação da recorrência discursiva^(12)^. Inicialmente, aplicou-se um questionário com questões sobre a idade, sexo, profissão na unidade, nível de escolaridade, pós-graduação na área de UTI, pós-graduação na área de CP, disciplinas ou cursos de CP durante a formação acadêmica e/ou profissional, atuação prévia em UTI, tempo de atuação profissional, tempo de atuação em UTI e tempo de experiência em CP. As entrevistas foram gravadas utilizando recursos do *Google Meet*.

### Análise dos dados

O conteúdo discursivo foi transcrito na íntegra, de forma separada, pela pesquisadora principal do projeto e por uma segunda pesquisadora, para maior validade do processo e posterior organização dos discursos. O corpus discursivo foi ajustado para submissão e processamento pelo programa IRaMuTeQ (*Interface de R para as Análises Multidimensionais de Textos e Questionários*). O software permite diferentes formas de tratamento e análise de dados textuais, como o cálculo da frequência de palavras (lexicografia básica) e análises multivariadas, como a classificação hierárquica descendente (CHD) e as análises de similitude^(13)^.

Para a análise estatística dos discursos, optou-se pelo método de CHD, que classifica os segmentos textuais de acordo com seus vocabulários, visando obter classes de Unidade de Contexto Elementar (UCE) que apresentam vocabulário semelhante entre si e diferente de outras classes^(13)^. Ressalta-se que foram consideradas para a análise somente as palavras que obtiveram resultado estatisticamente significativo, com valor de p<0,001. A partir dos pressupostos da TFVP, as categorias analíticas identificadas pelo método de CHD permitiram o reconhecimento de ações de cuidados e experiências dos profissionais entrevistados.

O movimento compreensivo e interpretativo concentrou-se nas categorias: “Ações multidisciplinares para o conforto no fim da vida” e “Cuidado paliativo no período pandêmico e a promoção do conforto emocional e espiritual”, conforme o Quadro 1. Os participantes foram identificados com siglas de acordo com sua categoria profissional e ordem de participação, para preservar o sigilo e a ética da pesquisa. A sigla “E” corresponde ao enfermeiro, “F” ao fisioterapeuta, “T” ao técnico de enfermagem, “P” ao psicólogo e “M” ao médico, com a numeração correspondente à ordem de entrevista dentro de cada categoria.

**Quadro 1:** Processo de redução das classes geradas pela Classificação Hierárquica Descendente e criação das categorias analíticas. Fortaleza, Ceará, Brasil, 2022

Análise Lexográfica			Classes (% do total)	Categorias segundo a TFVP (Teoria do Final de Vida Pacífico)
Palavra	X^2^	%		
Conseguir Conversar Médico Vez UTI Trabalhar Sair	32.98 29.95 28.52 25.67 25.11 24.75 15.21	61.90 100.00 46.15 47.06 62.5 100.00 58.33	1- Dificuldades da paliação no cenário pandêmico (17,16%)	Cuidado paliativo no período pandêmico e a promoção do conforto emocional e espiritual (42,2%)
Colocar Música Católico Família Dia Pedir Gostar Óleo da unção Consciente Ficar	62.33 57.04 40.34 34.23 32.09 26.22 24.20 23.96 21.84 16.23	73.33 100.00 100.00 44.44 83.33 71.43 80.00 100.00 62.50 34.62	4 - Conforto emocional e espiritual frente às dificuldades pandêmicas (11,27%)	
Visita Enfermaria Familiar Óbito Chorar Pessoa Contato Momento Liberar Celular Existir	45.56 38.86 36.12 25.65 25.65 20.38 20.38 19.29 19.14 19.14 16.73	100.00 69.23 69.23 100.00 100.00 71.43 71.43 50.00 100.00 100.00 62.50	6 - Aproximação entre os sujeitos no adoecer e no morrer no período pandêmico: a reintegração da espiritualidade e da individualidade dos sujeitos (13,73%)	
Cuidado Paliativo Ação Reiki Questão Massagem	83.29 81.32 20.40 16.66 15.22	71.88 73.33 100.00 45.83 100.00	2 - Atenção ao conforto espiritual (16,67%)	Ações multidisciplinares para o conforto no fim da vida (57,8%)
Máximo Tentar Paliativo Enfermagem Manter Gente Medida	39.00 30.18 29.31 28.89 24.46 17.40 16.73	80.00 66.67 46.43 43.75 63.64 47.06 62.50	3 - O esforço multiprofissional para o alívio do sofrimento físico (13,73%)	
Continuar Mudança de decúbito Mudo Cuidado Higiene Mudar Só Enfermagem	98.98 47.45 44.26 38.70 36.70 21.88 17.83 17.27	93.33 64.71 100.00 42.11 100.00 80.00 46.67 34.38	5 - Ausência de mudança na rotina de cuidados de enfermagem e atenção aos aspectos físicos do cuidado (12,25%)	
Fisioterapia Modo Aspiração Secreção Ventilador Desconfortável Tirar Condição	96.21 22.77 22.77 22.77 16.99 16.99 16.70 16.70	94.44 100.00 100.00 100.00 100.00 100.00 80.00 80.00	7 - Atenção aos aspectos físicos do cuidado e limitação no uso de medidas invasivas dolorosas (15,2%)	

*X^2^: Qui-quadrado.*

## RESULTADOS

Quanto aos aspectos sociais e profissionais dos participantes, 24 (77,4%) eram do sexo feminino. A idade média foi de 39,4 anos. Em relação à escolaridade, a maioria havia concluído a graduação (90,3%). Apenas dois (6,4%) participantes possuíam especialização na área de CP, e 20 profissionais (64,5%) relataram a inexistência de disciplinas de CP nos cursos de graduação ou pós-graduação, como demonstrado na Tabela 1. É importante ressaltar que, apesar da maioria dos sujeitos não ter especialização em CP ou formação específica na área durante os cursos formativos, eles informaram ter proximidade com a equipe especialista em CP devido à intensa atuação dessa equipe no período pré-pandêmico no hospital do estudo.

**Tabela 1:** Caracterização sociodemográfica dos sujeitos incluídos na pesquisa. Fortaleza, Ceará, Brasil, 2022

Característica dos participantes	n (%)
Gênero	
Feminino	24 (77,4)
Masculino	7 (22,5)
Idade (anos)	
Média±DP	39,38±6,1
Mínima	29
Máxima	54
Escolaridade máxima	
Nível técnico	3 (9,6)
Graduação	10 (32,2)
Especialização	8 (25,8)
Mestrado	8 (25,8)
Doutorado	5 (16,1)
Especialização em Cuidado Paliativo	
Sim	2 (6,4)
Não	29 (93,5)
Disciplina ou curso de Cuidado Paliativo na formação profissional	
Sim	11 (35,4)
Não	20 (64,5)

*DP: desvio padrão.*

Após o processamento do corpus no *software* de análise textual, obteve-se o aproveitamento de 91,5% do material submetido. A CHD possibilitou a identificação de sete classes, geradas pelo *software* (Figura 1).

**Figura 1:** Dendograma das classes fornecidas pelo *IRaMuTeQ*. Fortaleza, Ceará, Brasil, 2022

Procedeu-se à junção das classes dois, três, cinco e sete, que continham falas referentes à atenção dos profissionais ancoradas principalmente no pressuposto “ausência de dor” e, em menor quantidade, no pressuposto “estar em paz”, compondo 57,8% do conteúdo. Essa categoria foi nomeada “Ações multidisciplinares para o conforto no fim da vida”.

Posteriormente, realizou-se a junção das classes um, quatro e seis, considerando suas semelhanças, que continham falas representando as estratégias dos profissionais para superar as dificuldades da implementação do cuidado paliativo durante a pandemia. Essas falas também abrangiam os pressupostos de “experiência do conforto”, “dignidade e respeito”, “proximidade com pessoas importantes” e “estar em paz”. Essa segunda categoria de análise compôs 42,2% dos discursos e foi nomeada “Cuidado paliativo no período pandêmico e a promoção do conforto emocional e espiritual”.

Os resultados a seguir serão apresentados conforme as categorias emergentes após o movimento compreensivo.

### Ações multidisciplinares para o conforto no fim da vida

A compreensão desta categoria analítica demonstrou a predominância de ações de cuidado que priorizavam o pressuposto “ausência de dor”, revelado na preocupação dos profissionais em otimizar a utilização de medidas farmacológicas para o alívio de dores, com destaque para os participantes da categoria de enfermagem e fisioterapia.

*Olha, era conforto, conforto de analgesia, sabe?* (E08)

*As principais que a gente usa e usava na época era a questão de controle de dor, né? muito controle de dor.* (F03)

Alguns profissionais da equipe técnica de enfermagem relataram o uso de estratégias não farmacológicas e de terapias alternativas para o alívio de ansiedades e a promoção da espiritualidade dos pacientes, revelando a utilização do pressuposto “estar em paz” da TFVP, conforme demonstrado nos discursos a seguir.

*Fazia massagens. Utilizava o Reiki, fazia a oração* [...] *Eu dava muito o toque das mãos. Os pacientes mais debilitados emocionalmente, depressivos ou até mesmo com ansiedade, eu fazia muito o Reiki.* (T10)

### Cuidado paliativo no período pandêmico e a promoção do conforto emocional e espiritual

Esta categoria favoreceu a compreensão da dificuldade de inserção da equipe de CP no contexto de assistência ao paciente crítico com SARS-COV-2, para além das ações referidas.

*Não tinha cuidados paliativos. Quem fechava, mais ou menos, era o médico, o chefe da uti e a equipe médica da uti. Não tinha participação da equipe de cuidados paliativos na uti Covid não.* (E09)

Um dos motivos referidos para isso foi o aumento da demanda de trabalho da equipe especialista de CP.

[...] *a gente não conseguiu ter a participação de maneira tão frequente quanto nós gostaríamos da equipe de cuidados paliativos* [...]*. Porque a demanda foi muito grande em outros setores também do hospital.* (M04)

Uma outra causa relatada pelos profissionais para a diminuição da atuação da equipe de CP no setor foi a questão de limitação no uso de materiais de proteção individual, além das restrições devido ao risco de infecção.

*Eu acho que até pela dificuldade de acesso, no início. De acesso aos pacientes. Era complexo, por uma questão de material* [...]. (E02)

Além disso, o medo do desconhecido, devido ao adoecimento por uma condição clínica nova, acabou por gerar uma maior resistência dos profissionais em acionar a equipe de paliação.

*O fato de a doença ser muito nova, o paliativo quando era só Covid e não tinha outra coisa, quando a gente chamava, era já muito tardio. E muitas vezes, meio que a contragosto. Não existia uma concordância plena de todo mundo.* (M02)

No entanto, apesar de tais dificuldades, percebe-se nos discursos que a ausência da equipe especialista motivou os profissionais assistenciais das unidades a tomarem para a si a responsabilidade de fornecer o CP.

[...] *acaba que muitas vezes a gente, como equipe de uti, tinha que se encarregar totalmente dessa questão. Houve um pouco mais de autonomia nesse sentido. A gente tinha como fazer algumas intervenções mais precocemente com os familiares, uma vez que a gente conseguia perceber a irreversibilidade do quadro clínico do paciente, da gravidade.* (M04)

A compreensão do conteúdo discursivo dos participantes revela que apesar da dificuldade de inserção do CP em tempos de pandemia, a equipe multiprofissional manteve o esforço para o fornecimento de tal cuidado, através da utilização de estratégias embasadas nos princípios paliativistas.

As ações de CP mencionadas possibilitam a percepção de uma assistência que visava o alívio de sofrimentos gerados pela pandemia, através do fornecimento de ações baseadas no pressuposto de “experiência do conforto”, por meio da promoção do conforto emocional e espiritual, além de ações baseadas no pressuposto “proximidade com pessoas importantes”, a partir da promoção do contato entre paciente e família, para superação do isolamento social gerado durante este período.

[...] *A gente ia atender o paciente e fazia muito esse movimento de aproximação afetiva do paciente com a família, através de videochamada.* (P01)

*A gente viu realmente que trazia conforto e alívio, no sentido de deixar a família mais próxima. Acabava liberando para família vir visitar também, mesmo sendo Covid, e trouxe essa possibilidade, porque na hora que o paliativo vinha, a gente já pensava em deixar a família entrar.* (M02)

O respeito às crenças pessoais foi mencionado, com a inserção de rituais religiosos, conforme o pedido das famílias e dos pacientes, demonstrando a utilização do pressuposto “dignidade e respeito” e “estar em paz” elencados na TFVP.

*A gente respeitava muito a questão das crenças religiosas dos paciente* [...] *Tinha um médico que colocava na prescrição: água benta, 3 vezes ao dia. Óleo da unção, 2 vezes ao dia. Do jeito que a família pedia, ele colocava lá para colocar no paciente esses cuidados* [...] *Eu sou católica, mas eu tenho que respeitar a religião dos outros.* (E09)

Os discursos acima demonstram a utilização de estratégias para o fortalecimento de vínculos enfraquecidos e o fornecimento de ações para a reestruturação da saúde mental e espiritual dos pacientes em intenso sofrimento, além de adotarem um CP baseado na manutenção da dignidade dos sujeitos e o respeito às suas crenças, demonstrando a utilização prática dos pressupostos elencados na TFVP.

## DISCUSSÃO

Os resultados deste estudo refletem a complexidade do cuidado paliativo no contexto da pandemia de SARS-CoV-2 e as ações empreendidas pelos profissionais de saúde. O estudo demonstra, em primeiro lugar, a presença de diversas barreiras para a implementação do CP durante a pandemia. Dificuldades semelhantes são referidas em uma pesquisa realizada no Canadá, a qual demonstrou que as medidas de controle de infecção e a restrição quanto ao uso de EPIs e quanto ao número de profissionais nas UTI foram barreiras para a presença mais efetiva dos profissionais paliativistas nas unidades^(3)^, corroborando os achados do presente estudo.

Uma pesquisa realizada na Dinamarca, a qual comparou a taxa de referenciamento de pacientes para o serviço de CP no período pré e pós pandemia de SARS-CoV-2, encontrou uma diminuição no acionamento da equipe de cuidados paliativos. Tais achados, juntamente com os do presente estudo, trazem à tona a necessidade da gestão local dos serviços de saúde atuarem de modo a minimizar tais barreiras, através da destinação de recursos para propiciar a atuação paliativa, como a contratação de profissionais especialistas para atender à elevada demanda de pacientes com necessidades paliativas, além de investimento em ações de Educação Permanente dos profissionais assistenciais acerca do CP, a fim de possibilitar a manutenção da qualidade do cuidado oferecido a pacientes em situações de ameaça à vida^(14)^.

Apesar das barreiras impostas pela pandemia, o movimento compreensivo dos discursos possibilitou o entendimento de que a equipe multiprofissional das UTIs manteve um esforço para oferecer um cuidado focado no alívio do sofrimento dos pacientes com necessidades paliativas. O estudo demonstrou uma proximidade com os pressupostos da TFVP na assistência realizada pelos profissionais não especialistas em CP. Tal fornecimento do CP pelas equipes assistenciais é fato já recomendado pela Academia Nacional de Cuidados Paliativos (ANCP), reconhecendo, em especial, os profissionais da UTI como capazes de atender à maioria das demandas paliativas de seus clientes^(15)^. Entretanto, tal fato não exclui a necessidade da gestão local de garantir a presença de profissionais especialistas em CP nas unidades hospitalares, a fim de prestarem auxílio na condução de necessidades especiais e complexas dos pacientes.

Um estudo realizado em uma UTI do Estado da Bahia, o qual se embasou na TFVP como arcabouço teórico, os autores encontraram que todos os integrantes da equipe multiprofissional consideraram o alívio da dor como aspecto fundamental no cuidado ao paciente em paliação. Além disso, os participantes revelaram valorizar a aproximação da família durante o cuidado com o paciente em fase terminal de adoecimento^(16)^, indo ao encontro dos resultados encontrados no presente estudo.

Ao se considerar o cuidado com o paciente em dor, entretanto, deve-se levar em consideração o conceito atual de dor elencado pela Associação Internacional para o Estudo da Dor (IASP), no qual a dor é conceituada como uma experiência sensorial e emocional desagradável, tipicamente causada ou que lembre um dano tecidual real ou potencial. A associação reconhece o aspecto subjetivo da dor, a qual pode ser influenciada por aspectos físicos, psicológicos e sociais^(17)^. Dessa forma, os profissionais devem manter a atenção a aspectos para além da manifestação física do fenômeno doloroso, considerando sua complexidade e multifatorialidade.

Além do cuidado com a dor, a experiência do conforto, de forma multidimensional, apareceu com destaque nos discursos dos participantes. Para as teóricas da TFVP, a experiência do conforto envolve o alívio de quaisquer desconfortos, um estado de tranquilidade e contentamento ou qualquer coisa que torne a vida agradável^(8)^. Os discursos dos participantes demonstram a atenção para a questão do conforto através das preocupações com a analgesia, terapia posicional, evitar o uso de métodos dolorosos de forma frequente, bem como preocupações com oxigenioterapia e estética corporal. Além disso, o uso de terapias alternativas, como o Reiki, demonstra a visão para o conforto multidimensional, conforme a TFVP preconiza. Ademais, o uso de terapias alternativas e complementares é prática recomendada pelo Ministério da Saúde, o qual reconhece que estas abordagens buscam estimular mecanismos naturais para a restauração da saúde, através da visão ampliada do processo saúde-doença e da promoção de um cuidado humanizado, devendo ser estimulado pelos gestores de saúde^(18)^.

Por outro lado, o uso de práticas religiosas, bem como o fornecimento de ações para promoção da espiritualidade, demonstrada nos discursos dos participantes, demonstram a aproximação com os pressupostos de “dignidade e respeito”, bem como “estar em paz”. Estudo qualitativo baseado nos pressupostos da TFVP demonstrou que enfermeiros valorizam a espiritualidade como um recurso para a promoção da paz, através da inserção de práticas religiosas^(7)^.

Ademais, o pressuposto “proximidade com pessoas importantes” destacou-se de modo relevante, ao se compreender a preocupação dos profissionais em possibilitar a aproximação entre entes queridos e a superação do distanciamento social imposto pela pandemia. Para a TFVP, tal pressuposto envolve o sentimento de proximidade e de conectividade com outros indivíduos considerados importantes pelo paciente. Em uma perspectiva ampliada, significa possibilitar o envolvimento dos familiares na assistência^(8)^. Um estudo de revisão acerca do fornecimento do CP a pacientes graves no período pandêmico demonstrou que a maioria dos estudos apontam para a utilização de estratégias de aproximação entre paciente e família, principalmente através da realização de visitas virtuais, possibilitando amenizar o impacto do distanciamento social imposto durante o período de internamento^(19)^.

### Limitações do estudo

Este estudo apresenta algumas limitações que merecem ser discutidas. Em primeiro lugar, é importante mencionar o grande número de recusas por parte dos sujeitos convidados a participar da pesquisa. Isso ressalta a persistente complexidade envolvida na condução de pesquisas de natureza qualitativa, devido à necessidade de garantir a participação ativa e voluntária dos entrevistados.

Além disso, a pesquisa foi conduzida em um hospital terciário que dispõe de equipes especializadas na prestação de Cuidados Paliativos. Consequentemente, os sujeitos entrevistados possuem uma considerável experiência no campo dos Cuidados Paliativos, dada a natureza de suas atividades cotidianas. Essa expertise pode ter influenciado a abordagem e as ações relacionadas à paliação no contexto da pandemia, tornando esses resultados potencialmente não representativos da realidade encontrada em outros estabelecimentos de saúde no cenário nacional. Portanto, é fundamental considerar a possível variabilidade entre instituições de saúde que podem não ter o mesmo grau de especialização em Cuidados Paliativos, o que pode impactar na oferta e qualidade do cuidado paliativo fornecido aos pacientes com Covid-19.

### Contribuições para a área da Enfermagem, Saúde ou Política Pública

Acredita-se que o conhecimento acerca da condução da paliação por profissionais assistenciais não especialistas, durante a pandemia de *SARS-COV-2*, pode possibilitar a criação de estratégias para o enfrentamento de situações de catástrofes e de abalos no sistema de saúde futuras, com a manutenção da assistência paliativa a pacientes que enfrentam intenso sofrimento humano em tais cenários. À medida que os resultados deste estudo indicam que profissionais de diferentes áreas tiveram que se envolver diretamente na prestação de cuidados paliativos, eles também destacam a necessidade de integrar os Cuidados Paliativos nas políticas de saúde em todos os níveis. Isso pode envolver a criação de equipes interdisciplinares ou a revisão de políticas para garantir que pacientes com necessidades paliativas recebam assistência adequada, independentemente da situação de emergência.

## CONSIDERAÇÕES FINAIS

Nossos dados demonstram a necessidade de a gestão local dos serviços de saúde investir em ações para minorar as barreiras para a efetivação da assistência paliativa em períodos de abalo do sistema de saúde, com a garantia da presença de profissionais especialistas nas unidades, bem como por meio do investimento contínuo em ações de Educação Permanente acerca do CP. No entanto, apesar das barreiras impostas pela pandemia, a equipe assistencial das UTIs demonstrou a utilização de estratégias multidimensionais para um eficaz fornecimento de ações de alívio do sofrimento de pacientes com necessidades paliativas, com a promoção de um cuidado em proximidade com o preconizado pela Teoria do Fim de Vida Pacífico.

Dessa forma, compreende-se que a equipe não especialista seja capaz de fornecer um cuidado paliativo básico de forma adequada, sendo necessário, entretanto, a presença de profissionais paliativistas para a condução de necessidades especiais e complexas dos pacientes. Além disso, demonstra-se o valor das ações paliativas para um cuidado de saúde de qualidade e centrado no paciente.

## Data Availability

https://doi.org/10.48331/scielodata.NIZRCM
